# Intratumoral delivery of dendritic cells plus anti-HER2 therapy triggers both robust systemic antitumor immunity and complete regression in HER2 mammary carcinoma

**DOI:** 10.1136/jitc-2022-004841

**Published:** 2022-06-16

**Authors:** Ganesan Ramamoorthi, Krithika Kodumudi, Colin Snyder, Payal Grover, Hongtao Zhang, Mark I Greene, Amrita Basu, Corey Gallen, Doris Wiener, Ricardo L B Costa, Hyo S Han, Gary Koski, Brian J Czerniecki

**Affiliations:** 1 Clinical Science & Immunology Program, Moffitt Cancer Center, Tampa, Florida, USA; 2 Department of Pathology and Laboratory Medicine, University of Pennsylvania, Philadelphia, Pennsylvania, USA; 3 Department of Breast Oncology, Moffitt Cancer Center, Tampa, Florida, USA; 4 Biological Sciences, Kent State University, Kent, Ohio, USA

**Keywords:** immunotherapy, dendritic cells, CD4-positive T-lymphocytes, B-lymphocytes, natural killer T-cells

## Abstract

**Background:**

Human epidermal growth factor receptor 2 (HER2) targeted antibodies in combination with chemotherapy has improved outcomes of HER2 positive (pos) breast cancer (BC) but toxicity of therapy remains a problem. High levels of tumor-infiltrating lymphocytes are associated with increased pathologic complete responses for patients treated with neoadjuvant therapy. Here we sought to investigate whether delivery of intratumoral (i.t.) multiepitope major histocompatibility complex (MHC) class II HER2 peptides-pulsed type I polarized dendritic cells (HER2-DC1) in combination with anti-HER2 antibodies without chemotherapy could enhance tumor regression by increasing anti-HER2 lymphocyte infiltration into the tumor.

**Methods:**

BALB/c mice bearing orthotopic TUBO tumors, BALB/c mice bearing subcutaneous (s.c.) CT26 hHER2 tumors, or BALB-HER2/neu transgenic mice were all treated with i.t. or s.c. HER2-DC1, anti-HER2 antibodies, paclitaxel, T-DM1 or in combination. Immune response, host immune cells and effector function were analyzed using flow cytometry, interferon-γ ELISA and cytokine/chemokine arrays. The contributions of CD4^+^ and CD8^+^ T cells and antibody dependent cellular cytotoxicity (ADCC) were assessed using depleting antibodies and FcγR KO mice. Molecular changes were evaluated by immunohistochemistry and western blot.

**Results:**

HER2-DC1 combined with anti-HER2 antibodies delivered i.t. compared to s.c. induced complete tumor regression in 75–80% of treated mice, with increased tumor infiltrating CD4^+^ and CD8^+^ T, B, natural killer T cells (NKT) and natural killer cells, and strong anti-HER2 responses in all HER2^pos^ BC models tested. The therapy caused regression of untreated distant tumors. Labeled HER2-DC1 migrated prominently into the distant tumor and induced infiltration of various DC subsets into tumors. HER2-DC1 i.t. combined with anti-HER2 antibodies displayed superior antitumor response compared to standard chemotherapy with anti-HER2 antibodies. Lasting immunity was attained which prevented secondary tumor formation. The presence of CD4^+^ and CD8^+^ T cells and ADCC were required for complete tumor regression. In the HER2^pos^ BC models, HER2-DC1 i.t. combined with anti-HER2 antibodies effectively diminished activation of HER2-mediated oncogenic signaling pathways.

**Conclusions:**

HER2-DC1 i.t. with anti-HER2 antibodies mediates tumor regression through combined activation of T and B cell compartments and provides evidence that HER2-DC1 i.t. in combination with anti-HER2 antibodies can be tested as an effective alternative therapeutic strategy to current chemotherapy and anti-HER2 antibodies in HER2^pos^ BC.

**WHAT IS ALREADY KNOWN ON THIS TOPIC**

- HER2-directed therapeutics combined with chemotherapy have demonstrated benefit in about 50% of patients with human epidermal growth factor receptor 2 (HER2)-positive (pos) advanced breast cancer (BC), but incomplete response and significant toxicity from chemotherapy remains a problem.

**WHAT THIS STUDY ADDS**

- This study demonstrates that intratumoral delivery (i.t.) of HER2 peptide-pulsed type 1 polarized dendritic cell (HER2-DC1) plus anti-HER2 antibodies was more effective than standard chemotherapy combined with anti-HER2 antibodies and generated robust systemic antitumor immunity and causing regression of distant tumor sites and modulated HER2 oncogenic signaling pathways in HER2^pos^ BC.

**HOW THIS STUDY MIGHT AFFECT RESEARCH, PRACTICE AND/OR POLICY**

- Combination of HER2-DC1 i.t. with HER2 targeted antibodies while deescalating cytotoxic chemotherapies may improve complete responses in HER2^pos^ BC neoadjuvant therapy. A clinical trial is ongoing to test this combination therapy in the neoadjuvant setting for patients with HER2^pos^ BC.

## Introduction

Human epidermal growth factor receptor 2 (HER2) overexpression accounts for 20%–25% of breast cancers (BC), and it causes more aggressive disease associated with higher recurrence rate and metastatic spread.^1^ HER2-targeting monoclonal antibodies such as trastuzumab and pertuzumab used in combination with chemotherapy in a perioperative setting is the prevailing approach and was approved for patients with early stage and locally advanced HER2^pos^ BC.^2 3^ In addition to the primary role of blocking HER2-directed oncogenic signaling pathways, trastuzumab and pertuzumab have the capability to stimulate antitumor immune responses.^4^ Trastuzumab and pertuzumab can induce antibody-dependent cellular cytotoxicity (ADCC) by triggering FCγRIII activity on natural killer (NK) cells.^5^ In patients with HER2^pos^ metastatic BC, trastuzumab has been shown to induce anti-HER2 CD8^+^ T cell immune response with improved progression-free survival.^6^ Trastuzumab was also observed to induce CD4^+^ helper T cell-associated antitumor immunity in patients with early HER2^pos^ BC.^7^ Trastuzumab and pertuzumab increase internalization of HER2 and presentation of HER2-derived peptides on major histocompatibility complex (MHC) class I molecules, which drive antigen-specific cytotoxic T lymphocytes.^8 9^

Despite the fact that HER2-directed therapeutic approaches have improved outcomes in early stage HER2^pos^ BC, many patients remain at risk of relapse or death.^10 11^ Polychemotherapy regimen is a standard neoadjuvant treatment added to HER2-targeted antibodies for patients with HER2^pos^ BC, but some patients suffer from significant toxicity. Various hematologic, neurologic, cardiac, and cognitive morbidities have been reported in patients with HER2^pos^ BC undergoing chemotherapy combined with HER2-targeted agents.^12 13^ Consequently, de-escalated strategies (eg, chemo-sparing) are being developed. To illustrate, results of a single-arm phase 2 trial support treatment of patients with early-stage node-negative HER2^pos^ BC with only a single chemotherapy agent (ie, paclitaxel) combined with trastuzumab.^14^ Furthermore, the HER2-targeted antibody-drug conjugate trastuzumab emtansine (T-DM1) has demonstrated effective in incomplete responders to trastuzumab/pertuzumab/neoadjuvant chemotherapy and in patients with metastatic HER2^pos^ BC. It is currently being used as the standard of care in second line of treatment.^15^ Nevertheless, limited treatment benefits have been noted in a substantial fraction of patients with HER2^pos^ BC with advanced disease.^16^ In addition, it should be emphasized that treatment with T-DM1 has been associated with equivalent absolute risk of clinically relevant adverse events compared with chemotherapy-based treatment.^17^ Trastuzumab deruxtecan (DS-8201a) therapy has shown durable antitumor response in patients with metastatic HER2^pos^ BC who had been previously treated with T-DM1. However, pulmonary toxicity was observed in a substantial fraction of patients.^18^ All the above problems suggest that a more effective combination treatment approach is needed with focus on de-escalated strategies aiming to improve better risk-benefit ratio. There is growing interest in the use of immunotherapy combined with HER2-targeted antibodies to enhance the clinical response and overcome the severe toxic side effects of chemotherapy in patients with HER2^pos^ BC.^19^ This is particularly appealing as HER2-directed immunotherapies have been associated with favorable toxicity profile when compared with standard chemotherapy.^20^ Chemotherapy itself may drive immunogenic cell death and is associated with driving an immune response to cause tumor regression,^19^ thus driving positive immune responses in the tumor microenvironment (TME) may be a rational approach.

The immunosuppressive TME can exploit antitumor immune responses and inhibit CD4^+^ and CD8^+^ T cells, leading to eventual escape of HER2^pos^ BC cells from immune surveillance.^21^ Prominent efforts have been made in recent years to develop immunotherapies to aid HER2-targeted therapies, modulate the immunosuppressive TME, and improve clinical outcomes in patients with HER2^pos^ BC. Dendritic cells (DC) are an effective delivery tool to generate a tumor antigen-specific immune response that is specifically directed to cancer cells.^22^ Progressive loss of anti-HER2 Th1 immunity in patients with HER2^pos^ BC was correlated with poor treatment response and prognosis.^23^ An experimental HER2 peptide-pulsed type 1 polarized dendritic cell (HER2-DC1) vaccine strongly restored anti-HER2 Th1 immune response in patients with both HER2^pos^ ductal carcinoma in situ and early HER2^pos^ invasive BC and improved pathologic complete responses (pCR).^23 24^ The canonical Th1 cytokine interferon-γ (IFN-γ) plays an important role in innate and adoptive immune responses, supporting its value as a mediator of effective antitumor immunity. Evidence exists that Th1 cytokines including IFN-γ can mediate HER2 degradation via the ubiquitin proteasomal pathway and also via caspase 3 in HER2^pos^ BC cells.^25 26^ IFN-γ also enhances expression of MHC class I/II and programmed death ligand-1 (PD-L1) on tumor cells, leading to recognition by immune cells.^27^ More recently, we showed that treatment of Th1 cytokines in combination with trastuzumab and pertuzumab synergistically increased tumor senescence and apoptosis via STAT1 signaling in HER2^pos^ BC cells.^28^ Notably, HER2-DC1 subcutaneous (s.c.) delivery in combination with anti-HER2/neu antibodies delayed tumor growth and improved survival in a HER2^pos^ BC mouse model.^29^ Studies have shown that intratumoral (i.t.) delivery of tumor antigen-pulsed DC can enhance the efficacy of targeted agents, increase tumor infiltration of CD4^+^ and CD8^+^ T cells and prolong antitumor immunity in patients with advanced stage solid tumor.^30 31^ Recently, DC i.t. combination with local radiotherapy has been observed to increase tumor antigen specific CD8^+^ T cell infiltration in poorly immunogenic tumor models.^32^

In the present study, we aimed to investigate the efficacy of HER2-DC1 i.t. delivery in combination with anti-HER2 antibodies and whether this combination approach could replace standard chemotherapy and drive enduring antitumor immunity in a HER2^pos^ BC preclinical model. We show that HER2-DC1 i.t. combined with anti-HER2 antibodies treatment was more effective than HER2-DC1 s.c. combined with anti-HER2 antibodies. Strikingly, in a clinically relevant HER2^pos^ BC model, HER2-DC1 i.t. combined with anti-HER2 antibodies treatment showed a superior antitumor response compared with standard chemotherapy combined with anti-HER2 antibodies. We have also revealed a role for CD4^+^ and CD8^+^ T cells and ADCC in combination treatment induced tumor regression in a HER2^pos^ BC model. HER2-DC1 i.t. combined with anti-HER2 antibodies treatment more effectively modulated HER2 oncogenic signaling pathways in a HER2^pos^ BC model. Furthermore, HER2-DC1 i.t. combined with anti-HER2 antibodies treatment effectively attenuated the growth of untreated distant HER2^pos^ tumors, highlighting its potential in generating systemic antitumor immunity in HER2^pos^ BC. Our study suggests new approaches to improve HER2^pos^ BC treatment by combining targeted antibodies with active immunotherapy while de-escalating cytotoxic chemotherapies.

## Methods

The compete experimental protocols are described in online supplemental material.

10.1136/jitc-2022-004841.supp1 Supplementary data

## Results

### HER2-DC1 s.c. and anti-HER2 antibodies combination treatment

**Figure 1:**
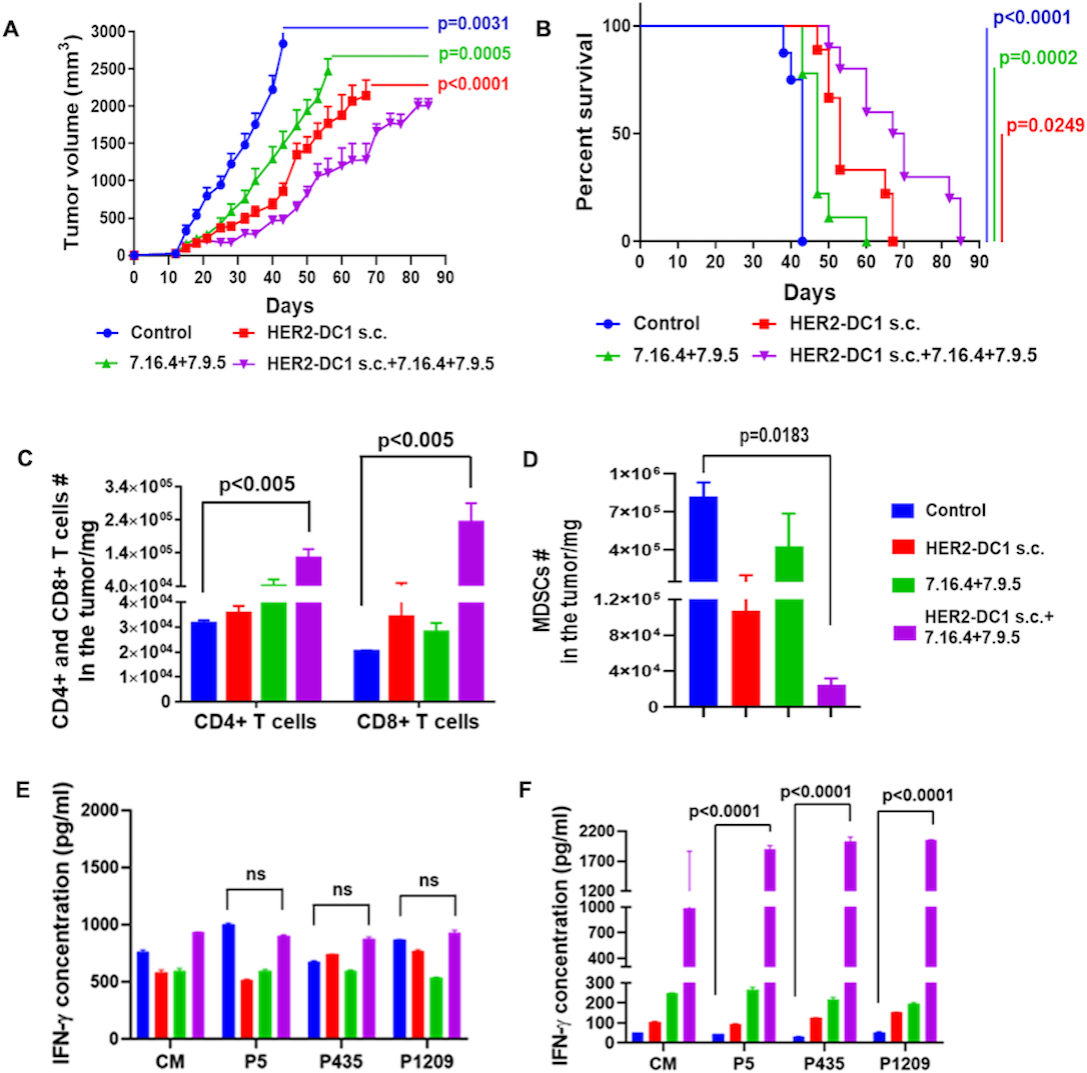
Antitumor efficacy of HER2-DC1 s.c. in combination with anti-HER2 antibodies treatment and host immune response in HER2^pos^ BC model. (A) Wild-type BALB/c mice were injected with 3×10^4^ TUBO cells orthotopically into the MFP. After tumor establishments, mice were treated with HER2-DC1 s.c. (1×10^6^ cells, s.c., weekly twice for 3 weeks) or anti-HER2 antibodies (clones 7.16.4: 50 µg, 7.9.5: 50 µg, i.p. injection, weekly once) or combination of both. Tumor growth was monitored two times a week (n=8). (B) Survival curve (n=8). Mean±SEM. Control versus HER2-DC1 s.c. +7.16.4+7.9.5 (p=0.0031) in (A) and (p<0.0001) in (B), HER2-DC1 s.c. versus HER2-DC1 s.c. +7.16.4+7.9.5 (p<0.0001) in (A) and (p=0.0249) in (B) and 7.16.4+7.9.5 versus HER2-DC1 s.c. +7.16.4+7.9.5 (p<0.0005) in (A) and (p=0.0002) in (B). (C, D) On day 36 after completion of treatments, tumors were excised, single cell suspensions were prepared and stained for CD4^+^ and CD8^+^ T cells and myeloid-derived suppressor cells, and then analyzed by flow cytometry. (E) Tumor draining lymph nodes and non-draining lymph nodes were collected from the experimental groups. Then, co-cultured with or without HER2-DC1 individually pulsed with p5, p435 and p1209 for 72 hours. IFN-γ secretion was measured in the culture supernatant using IFN-γ ELISA. (F) Splenocytes were re-stimulated with p5, p435 and p1209 rHER2/neu peptides. IFN-γ secretion was measured in the culture supernatant by ELISA. Control versus HER2-DC1 s.c. +7.16.4+7.9.5 (p<0.0001) in (F). BC, breast cancer; CM, culture medium; HER2, human epidermal growth factor receptor 2; HER2-DC1, HER2 peptide-pulsed type 1 polarized dendritic cell; IFN, interferon; i.p., intraperitoneal; MFP, mammary fat pad; s.c., subcutaneous.

To examine the efficacy of HER2-DC1 s.c. combined with anti-HER2 antibodies treatment and host immune response in HER2^pos^ BC, we utilized the HER2^pos^ TUBO tumor model. After tumor establishment, mice were treated with HER2-DC1 s.c., anti-HER2 antibodies, or in combination. HER2-DC1 s.c. combined with anti-HER2 antibodies treatment significantly delayed tumor growth and improved survival compared with HER2-DC1 s.c. alone or anti-HER2 antibodies alone (figure 1A, B). Next, changes in the level of host immune cells after completion of treatments were examined on day 36 by flow cytometry. Increased level of tumor infiltrating CD4^+^ and CD8^+^ T cells and decreased level of myeloid-derived suppressor cells (MDSC) were observed in the tumors of the combination treatment group, compared with monotherapy (figure 1C, D). As shown in figure 1E, no significant difference in the secretion of IFN-γ was observed after co-culturing individual peptide-pulsed HER2-DC1 with tumor draining lymph nodes (TDLNs) and non-draining lymph nodes (NDLNs) from the HER2-DC1 s.c., anti-HER2 antibodies or HER2-DC1 s.c. in combination with anti-HER2 antibodies treatment groups. Thus, similar levels of anti-HER2 Th1 immunity existed. We observed significantly increased IFN-γ secretion on restimulation of splenocytes from the HER2-DC1 s.c. combined with anti-HER2 antibodies treatment group with HER2 peptides p5, p435 and p1209, when compared with control (figure 1F).

### HER2-DC1 i.t. combination with anti-HER2 antibodies treatment enhance antitumor activity

**Figure 2:**
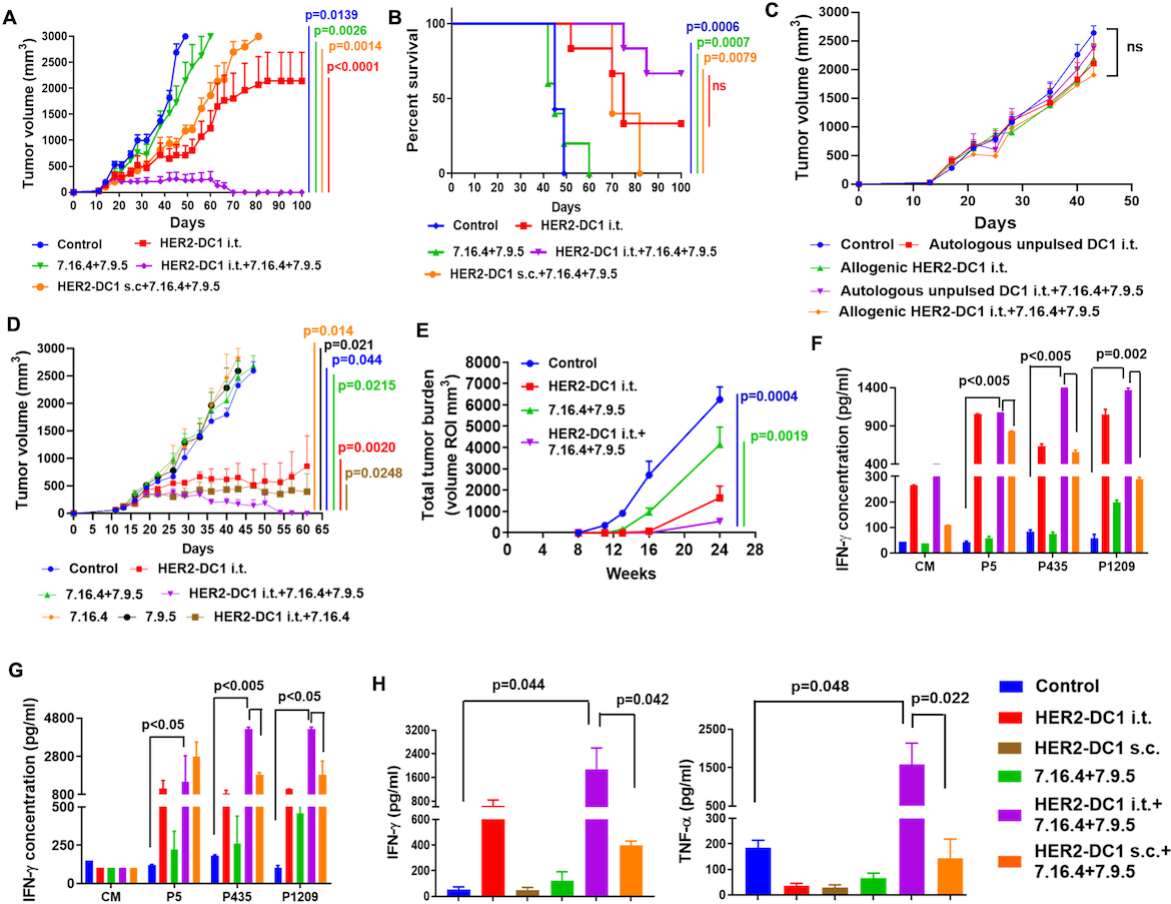
HER2-DC1 i.t. in combination with anti-HER2 antibodies treatment eradicates HER2^pos^ BC. (A) After TUBO tumor establishment, mice were treated as described in methods. Tumor growth was monitored two times a week (n=8). (B) Survival curve (n=8). Mean±SEM. Control versus HER2-DC1 i.t.+7.16.4+7.9.5 (p=0.0139) in (A) and (p=0.006) in (B), HER2-DC1 i.t. versus HER2-DC1 i.t.+7.16.4+7.9.5 (p<0.0001) in (A) and (ns) in (B), 7.16.4+7.9.5 versus HER2-DC1 i.t.+7.16.4+7.9.5 (p=0.0026) in (A) and (p=0.0007) in (B) and HER2-DC1 s.c. +7.16.4+7.9.5 versus HER2-DC1 i.t.+7.16.4+7.9.5 (p=0.0014) in (A) and (p=0.0079) in (B). (C) Tumor growth curves of BALB/c mice bearing TUBO tumors treated with autologous unpulsed DC1 i.t. alone, allogenic HER2-DC1 i.t. alone and autologous unpulsed DC1 i.t. or allogenic HER2-DC1 i.t. combined with anti-HER2 antibodies 7.16.4 and 7.9.5. Tumor growth was monitored two times a week (n=6–8). (D) BALB/c mice bearing TUBO tumors were treated with HER2-DC1 i.t. alone, anti-HER2 antibodies (two clones 7.16.4 and 7.9.5), clone 7.16.4 alone, clone 7.9.5 alone, HER2-DC1 i.t. in combination with both anti-HER2 antibodies (7.16.4 and 7.9.5) and HER2-DC1 i.t. in combination with single clone 7.16.4. Tumor growth was monitored two times a week (n=8). Mean±SEM. p=0.044, control versus HER2-DC1 i.t.+7.16.4+7.9.5; p=0.014, 7.16.4 versus HER2-DC1 i.t.+7.16.4+7.9.5; p=0.021, 7.9.5 versus HER2-DC1 i.t.+7.16.4+7.9.5; p=0.0215, 7.16.4+7.9.5 versus HER2-DC1 i.t.+7.16.4+7.9.5; p=0.0020, HER2-DC1 i.t. versus HER2-DC1 i.t.+7.16.4+7.9.5; p=0.0248, HER2-DC1 i.t.+7.16.4 versus HER2-DC1 i.t.+7.16.4+7.9.5. (E) Tumor growth in BALB-HER2/neu transgenic mice treated with HER2-DC1 i.t. alone or anti-HER2 antibodies (7.16.4 and 7.9.5) alone or combination of both (n=8). Spontaneous tumor growth in the mammary glands of experimental mice were monitored by MRI. Mean±SEM. p=0.0004, control versus HER2-DC1 i.t.+7.16.4+7.9.5; p=0.0019, 7.16.4+7.9.5 versus HER2-DC1 i.t.+7.16.4+7.9.5. (F) Tumor draining lymph nodes and non-draining lymph nodes were excised from the experimental groups and then, co-cultured with or without HER2-DC1 individually pulsed with p5, p435 and p1209 for 72 hours. IFN-γ secretion was measured in the culture supernatant using IFN-γ ELISA. (G) Splenocytes from the experimental mice were re-stimulated with p5, p435 and p1209 rHER2/neu peptides. Culture supernatant was collected and analyzed for IFN-γ secretion using ELISA. (H) Serum level of Th1 cytokines IFN-γ and TNF-α in the experiment groups were analyzed by Th cytokine flow cytometry array. The results were shown as mean±SEM of at least three independent experiments. BC, breast cancer; CM, culture medium; DC, dendritic cell; HER2, human epidermal growth factor receptor 2; HER2-DC1, HER2 peptide-pulsed type 1 polarized dendritic cell; IFN, interferon; i.t., intratumoral; ns, not significant; s.c., subcutaneous; TNF, tumor necrosis factor.

Next, we investigated whether HER2-DC1 i.t. combined with anti-HER2 antibodies treatment show superior antitumor effects compared with HER2-DC1 s.c. combined with anti-HER2 antibodies in the HER2^pos^ TUBO tumor model. We observed that HER2-DC1 i.t. combined with anti-HER2 antibodies treatment showed enhanced antitumor effects with complete tumor regression in 75% of the treated mice and prolonged survival compared with HER2-DC1 s.c. and anti-HER2 antibodies combination treatment (figure 2A, B). Importantly, mice with tumor regression were immune and rejected secondary TUBO tumor challenge (online supplemental figure 2). Pulsing with immunogenic multiepitope MHC class II HER2 peptides and the generation of a host specific anti-HER2 immune response was critical for the tumor regression efficacy of HER2-DC1 i.t. combined with anti-HER2 antibodies treatment, which was supported by the failure of controlling HER2^pos^ TUBO tumor growth after autologous unpulsed DC1 i.t. or allogenic HER2-DC1 i.t. combined with anti-HER2 antibodies treatment (figure 2C). Next, we investigated whether HER2-DC1 i.t. in combination with single anti-HER2 antibody clone is sufficient for the enhanced antitumor activity or required both anti-HER2 antibodies in the HER2^pos^ TUBO tumor model. Combination treatment of HER2-DC1 i.t. with single anti-HER2 antibody 7.16.4 clone showed a delay in tumor growth, but only induced complete tumor regression in 40% of treated mice (figure 2D). Interestingly, an enhanced antitumor response with complete tumor regression was observed in 80% of the mice treated with HER2-DC1 i.t. in combination with both anti-HER2 antibodies 7.16.4 and 7.9.5 (figure 2D). BALB-HER2/neuT mice that received HER2-DC1 i.t. and anti-HER2 antibodies (7.16.4 and 7.9.5) combination treatment also showed significant delay in tumor growth (figure 2E).

Anti-HER2 Th1 immune response was evaluated by co-culturing TDLNs and NDLNs with DC1 pulsed with HER2 peptides p5, p435 and p1209 individually. As shown in figure 2F, a significant increase in the level of IFN-γ secretion was observed after co-culturing individual peptide-pulsed HER2-DC1 with TDLNs and NDLNs from the HER2-DC1 i.t. combined with anti-HER2 antibodies treatment group, compared with the HER2-DC1 s.c. combined with anti-HER2 antibodies group. Similarly, restimulation of splenocytes from the HER2-DC1 i.t. combined with anti-HER2 antibodies treatment group with HER2 peptides p435 and p1209 showed a higher level of IFN-γ secretion compared with the HER2-DC1 s.c. combined with anti-HER2 antibodies group (figure 2G). Significantly increased serum levels of Th1 cytokines IFN-γ and tumor necrosis factor (TNF)-α were also observed in the HER2-DC1 i.t. and anti-HER2 antibodies combination treatment group compared with the HER2-DC1 s.c. combined with anti-HER2 antibodies treatment group (figure 2H). In addition, increased serum levels of other Th cytokines and chemokines were observed in the HER2-DC1 i.t. in combination with anti-HER2 antibodies treated group, and in immune mice that rejected secondary TUBO tumor challenge (online supplemental figures 1 and 2). Taken together, these data suggest that HER2-DC1 i.t. combined with anti-HER2 antibodies treatment induces a strong anti-HER2 Th1 immune response and enhances tumor regression activity in HER2^pos^ BC.

### HER2-DC1 i.t. combined with anti-HER2 antibodies treatment requires CD4^+^ and CD8^+^ T cells

**Figure 3:**
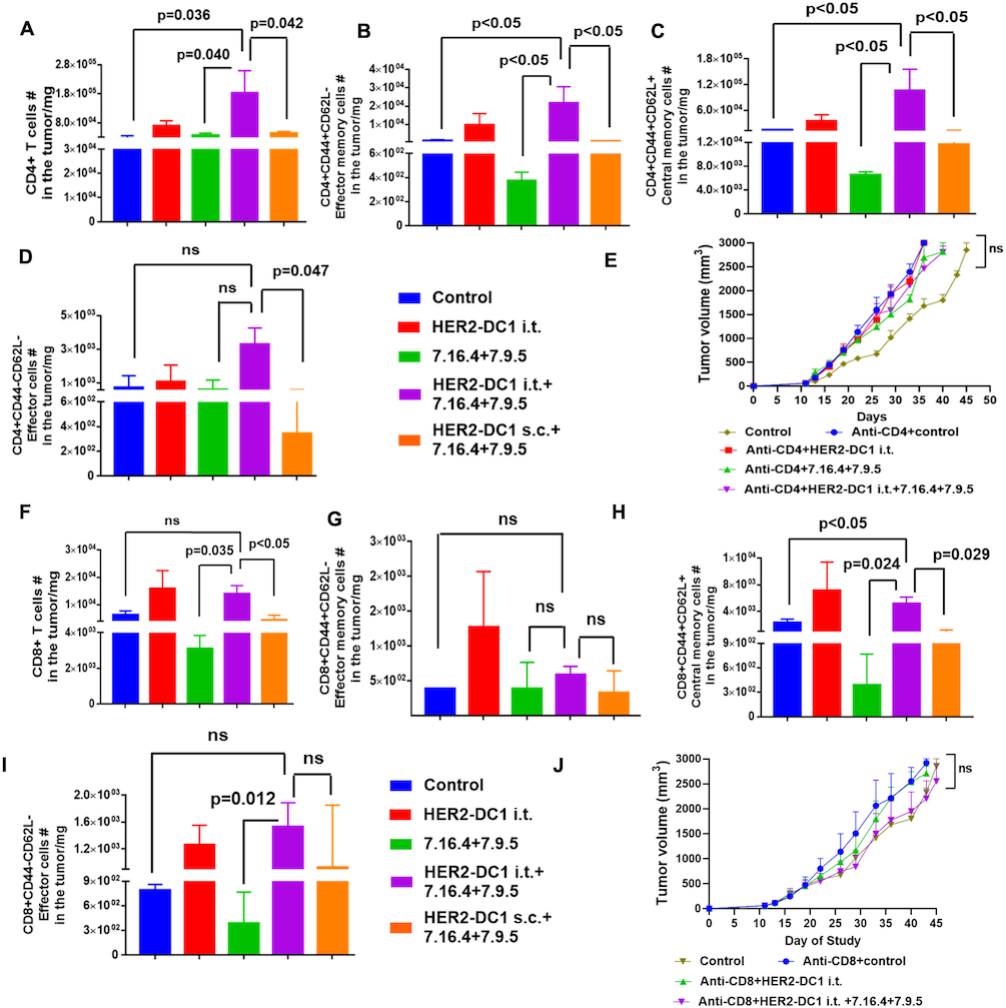
HER2-DC1 i.t. and anti-HER2 antibodies combination treatment induced tumor regression required CD4^+^ and CD8^+^ T cells. (A) On day 60 after completion of treatments, level of tumor infiltrating CD4^+^ T cells in the experimental groups was analyzed by flow cytometry. (B–D) The levels of CD4^+^CD44^+^CD62L^–^ effector memory cells, CD4^+^CD44^+^CD62L^+^ central memory cells and CD4^+^CD44^–^CD62L^–^ effector cells among the tumor infiltrated CD4^+^ T cells were analyzed by flow cytometry. (E) BALB/c mice were injected with CD4^+^ depleting antibody 3 days prior to TUBO induction and continued two time a week until the endpoint. After TUBO tumor establishments, mice were treated with HER2-DC1 i.t. alone or anti-HER2 antibodies (7.16.4 and 7.9.5) alone or combination of both (n=8). Mean±SEM. (F) Tumor infiltrating CD8^+^ T cells in the experimental groups on day 60 after completion of treatments. (G–I) The levels of CD8^+^CD44^+^CD62L^–^ effector memory cells, CD8^+^CD44^+^CD62L^+^ central memory cells and CD8^+^CD44^–^CD62L^–^ effector cells among the tumor infiltrated CD8^+^ T cells were assessed by flow cytometry. Results were shown as mean±SEM of at least three independent experiments. (J) BALB/c mice were administered with CD8 depleting antibody 3 days prior to TUBO induction and continued twice a week. TUBO tumor bearing mice were treated with treated with HER2-DC1 i.t. alone or anti-HER2 antibodies (7.16.4 and 7.9.5) alone or combination of both (n=8). Mean±SEM. HER2, human epidermal growth factor receptor 2; HER2-DC1, HER2 peptide-pulsed type 1 polarized dendritic cell; i.t., intratumoral; ns, not significant; s.c., subcutaneous.

The level of tumor infiltrating CD4^+^ T cells was evaluated after completion of all combination treatments. The flow cytometry gating strategy for lymphoid immune cell phenotype is shown in online supplemental figure 3. The HER2-DC1 i.t. combined with anti-HER2 antibodies treatment group had a higher number of tumor infiltrating CD4^+^ T cells, compared with the HER2-DC1 s.c. combined with anti-HER2 antibodies treatment group (figure 3A). Next, we examined the phenotypic status of tumor infiltrating CD4^+^ T cells. As shown in figure 3B–D, increased levels of CD4^+^CD44^+^CD62L^–^ effector memory cells, CD4^+^CD44^+^CD62L^+^ central memory cells and CD4^+^CD44^–^CD62L^–^ effector cells were observed in tumors from the HER2-DC1 i.t. combined with anti-HER2 antibodies group, compared with the HER2-DC1 s.c. combined with anti-HER2 antibodies group. To test whether CD4^+^ T cells play a role in HER2-DC1 i.t. in combination with anti-HER2 antibodies treatment mediated antitumor activity, a CD4^+^ T cell depletion experiment was performed. We observed mice that were depleted of CD4^+^ T cells failed to respond to HER2-DC1 i.t. combined with anti-HER2 antibodies treatment (figure 3E).

HER2-DC1 i.t. combined with anti-HER2 antibodies treatment was able to enhance tumor infiltration of CD8^+^ T cells compared with HER2-DC1 s.c. combined with anti-HER2 antibodies treatment (figure 3F). Next, we examined the phenotypic status of tumor infiltrating CD8^+^ T cells. Tumors from the HER2-DC1 i.t. combined with anti-HER2 antibodies treatment group had a significantly increased level of CD8^+^CD44^+^CD62L^+^ central memory cells with no differences in CD8^+^CD44^+^CD62L^–^ effector memory cells and CD8^+^CD44^–^CD62L^–^ effector cells when compared with HER2-DC1 s.c. combined with anti-HER2 antibodies treatment (figure 3G–I). Next, the role of CD8^+^ T cells were examined, and we found that HER2-DC1 i.t. in combination with anti-HER2 antibodies treatment failed to control TUBO tumor growth in the absence of CD8^+^ T cells (figure 3J). These data suggest that CD4^+^ and CD8^+^ T cells are critical for tumor regression induced by HER2-DC1 i.t. and anti-HER2 antibodies combination treatment in HER2^pos^ BC.

### HER2-DC1 i.t. in combination with anti-HER2 antibodies treatment attenuates growth of distant untreated tumors

**Figure 4:**
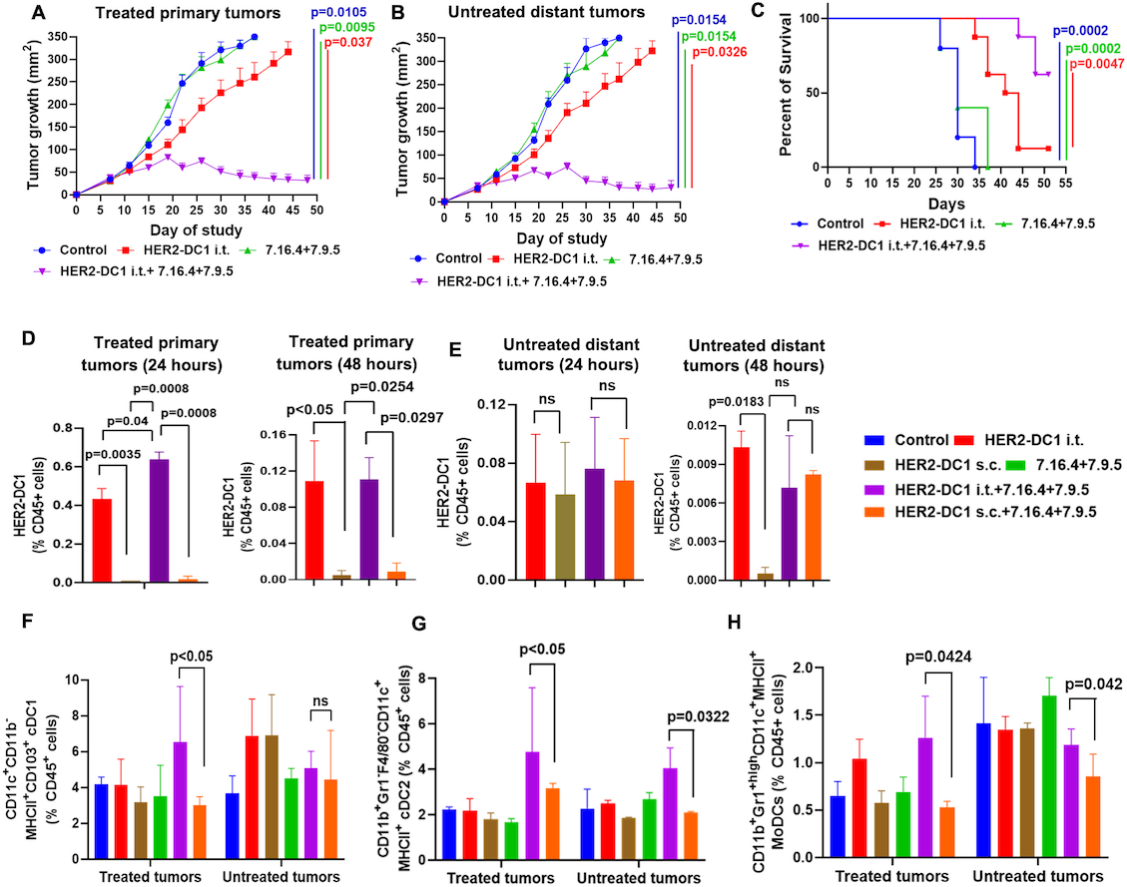
HER2-DC1 i.t. in combination with anti-HER2 antibodies treatment induce systemic antitumor immunity in HER2^pos^ BC model. (A) Tumor growth curves of treated primary tumors, (B) untreated distant tumors and (C) survival curves in TUBO bilateral tumor model received different treatments as indicated (n=8). Control versus HER2-DC1 i.t.+7.16.4+7.9.5 (p=0.0105) in (A), (p=0.0154) in (B) and (p=0.0002) in (C). HER2-DC1 i.t. versus HER2-DC1 i.t.+7.16.4+7.9.5 (p=0.037) in (A), (p=0.0326) in (B) and (p=0.0047) in (C). 7.16.4+7.9.5 versus HER2-DC1 i.t.+7.16.4+7.9.5 (p=0.0095) in (A), (p=0.0154) in (B) and (p=0.0002) in (C). (D) The frequency of CellTrace Violet labeled HER2-DC1 in the treated primary tumors at 24 and 48 hours. (E) The level of migrated CellTrace Violet labeled HER2-DC1 into the untreated distant tumors at 24 and 48 hours. (F–H) The levels of cDC1, cDC2 and MoDCs in the treated primary tumors and the untreated distant tumors were evaluated by flow cytometry. Mean±SEM. BC, breast cancer; cDC1, conventional DC1; cDC2, conventional DC2; DC, dendritic cell; HER2, human epidermal growth factor receptor 2; HER2-DC1, HER2 peptide-pulsed type 1 polarized dendritic cell; i.t., intratumoral; MoDCs, monocytic DCs; ns, not significant; s.c., subcutaneous; MHC, major histocompatibility complex.

We next investigated whether HER2-DC1 i.t. treatment combined with anti-HER2 antibodies would also trigger a systemic antitumor immunity by using a bilateral TUBO tumor model. HER2-DC1 i.t. was delivered in the primary tumors with or without anti-HER2 antibodies (7.16.4 and 7.9.5) and the growth of untreated distant tumors was monitored. Anti-HER2 antibodies treatment alone had no effect on the growth of primary and distant tumors while HER2-DC1 i.t. alone showed minimal antitumor effect on delaying both primary and distant tumors (figure 4A, B). Interestingly, HER2-DC1 i.t. combined with anti-HER2 antibodies (7.16.4 and 7.9.5) treatment significantly attenuated primary tumor and distant tumor growth and improved survival (figure 4A–C).

We then evaluated if the combination treatment-induced systemic antitumor immunity may have been facilitated by trafficking of injected HER2-DC1 into the untreated distant tumors. The flow cytometry gating strategy for identifying CellTrace Violet-labeled HER2-DC1 is shown in online supplemental figure 4. The frequency of CellTrace Violet labeled HER2-DC1 in the treated primary tumors was higher in the HER2-DC1 i.t. alone and the HER2-DC1 i.t. combined with anti-HER2 antibodies treatment groups at 24 and 48 hours (figure 4D), with only 2%–8% being apoptotic cells (online supplemental figure 5A, B). However, we did not observe CellTrace Violet-labeled HER2-DC1 trafficking into the primary tumors from the HER2-DC1 s.c. alone or HER2-DC1 s.c. combined with anti-HER2 antibodies groups (figure 4D). In the untreated distant tumors, a modest increase in the frequency of CellTrace Violet-labeled HER2-DC1 with no apoptotic cells was observed in the HER2-DC1 i.t. alone and HER2-DC1 i.t. combined with anti-HER2 antibodies groups at 24 and 48 hours (figure 4E, online supplemental figure 5C, D). Although we observed migration of CellTrace Violet-labeled HER2-DC1 into the untreated distant tumors in the HER2-DC1 s.c. alone and HER2-DC1 s.c. combined with anti-HER2 antibodies groups, 25%–45% of HER2-DC1 cells were apoptotic (figure 4E, online supplemental figure 5C, D). In addition, migration of HER2-DC1 into the TDLNs was observed in all groups with minimal changes in the percentage of apoptotic cells (online supplemental figure 6A–C).

Next, we evaluated the i.t. DC subsets in TME of treated and untreated tumors. The flow cytometry gating strategy for identifying i.t. DC subsets is shown in online supplemental figure 7. As shown in figure 4F, H, an increase in the level of conventional DC1 (cDC1) and monocytic DCs (MoDCs) was observed in the treated primary tumors from the HER2-DC1 i.t. combined with anti-HER2 antibodies group when compared with the HER2-DC1 s.c. combined with anti-HER2 antibodies group. Interestingly, both the treated primary tumors and the untreated distant tumors from the HER2-DC1 i.t. combined with anti-HER2 antibodies treatment group had a significantly increased level of cDC2 compared with the HER2-DC1 s.c. combined with anti-HER2 antibodies group (figure 4G). The frequency of tumor associated macrophages in the treated primary tumors and the untreated distant tumors were unchanged in both combination treatment groups (online supplemental figure 6D). Together, these results indicate that combination of HER2-DC i.t. and anti-HER2 antibodies increases the frequency of DC subsets in the TME, generates systemic antitumor immunity, and has the potential to attenuate the growth of distant tumors in HER2^pos^ BC.

### HER2-DC1 i.t. combined with anti-HER2 antibodies treatment enhances tumor infiltration of B, NKT and NK cells

**Figure 5:**
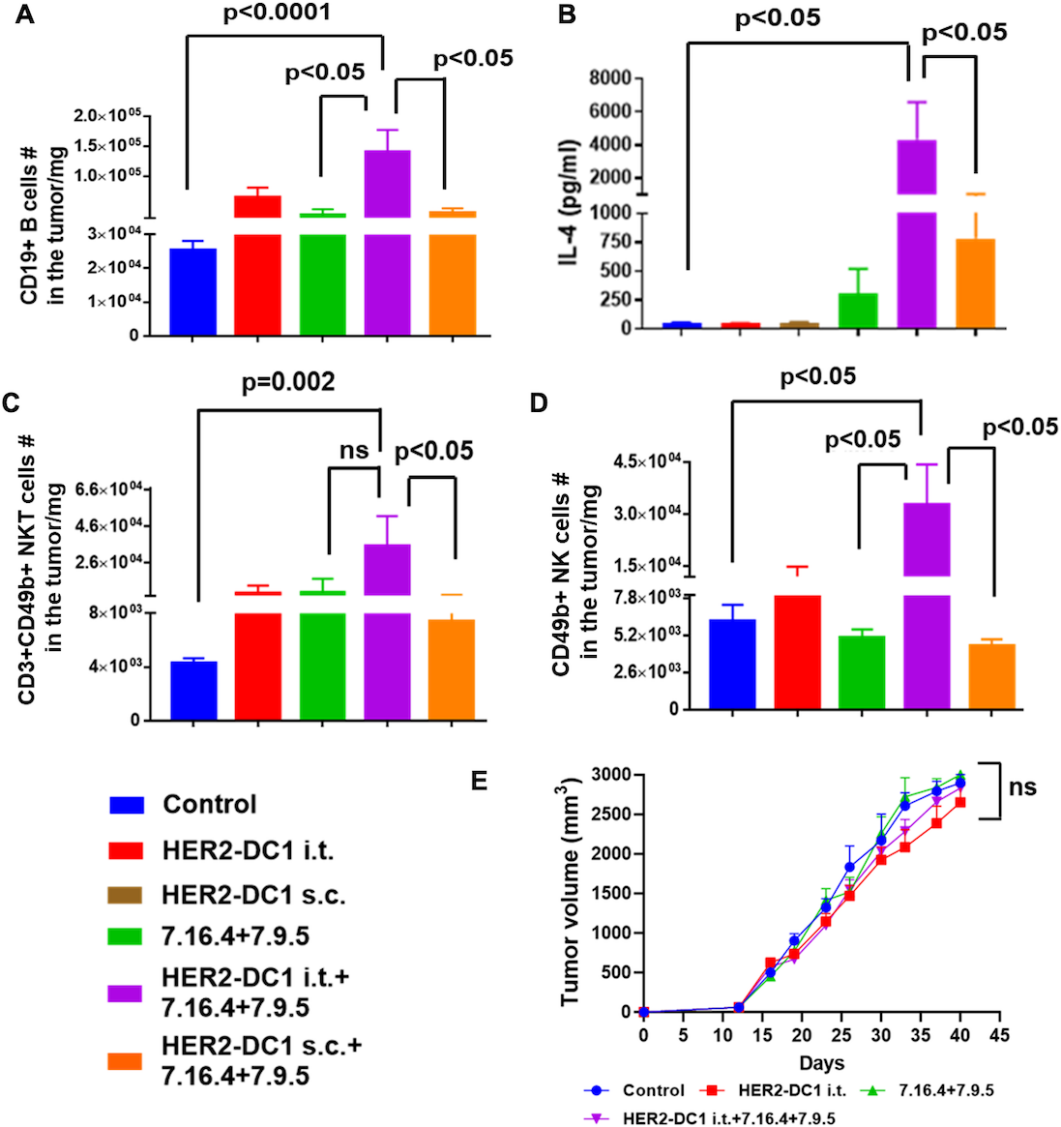
HER2-DC1 i.t. in combination with anti-HER2 antibodies treatment induces B, NKT and NK cells tumor infiltration and antibody-dependent cellular cytotoxicity. (A) Tumors from the experimental mice were collected on day 60 after completion of treatments, single cell suspensions were stained for CD19+ B cells as described in the methods and analyzed using flow cytometry. (B) Serum level of IL-4 in the experiment groups was analyzed by Th cytokine flow cytometry array. (C) Level of tumor infiltrating CD3+CD49 b (DX-5)+double positive NKT cells. (D) Tumor infiltrating level of CD49b (DX-5)+NK cells. Results were shown as mean±SEM of at least three independent experiments. (E) FcγR KO mice were injected with 3×10^4^ TUBO cells orthotopically into the MFP. After tumor establishment, mice were treated with HER2-DC1 i.t. alone or anti-HER2 antibodies (7.16.4 and 7.9.5) alone or combination of both as described in methods. Tumor growth was monitored two times a week (n=8). Mean±SEM. HER2, human epidermal growth factor receptor 2; HER2-DC1, HER2 peptide-pulsed type 1 polarized dendritic cell; IL, interleukin; i.t., intratumoral; MFP, mammary fat pad; NK, natural killer; ns, not significant; s.c., subcutaneous; NKT, natural killer T cells.

In addition to the level of tumor infiltrating CD4^+^ and CD8^+^ T cells, we also evaluated B cell, NKT cell and NK cells infiltration after HER2-DC1 i.t. and anti-HER2 antibodies combination treatment. As shown in figure 5A, significantly increased accumulation of B cells was observed in the tumors from the HER2-DC1 i.t. and anti-HER2 antibodies combination treatment group, compared with the HER2-DC1 s.c. and anti-HER2 antibodies combination treatment group. In addition, a significant increase in the serum level of interleukin (IL)-4, an important cytokine responsible for activation of mature B cells,^33^ was observed in the HER2-DC1 i.t. combined with anti-HER2 antibodies treatment group compared with the HER2-DC1 s.c. combined with anti-HER2 antibodies group (figure 5B). Next, increased levels of NKT cells and NK cells were observed in the tumors of mice that received HER2-DC1 i.t. and anti-HER2 antibodies combination treatment compared with those that received HER2-DC1 s.c. and anti-HER2 antibodies combination treatment (figure 5C, D). The flow cytometry gating strategy for myeloid cells is shown in online supplemental figure 8. HER2-DC1 i.t. or HER2-DC1 s.c. in combination with anti-HER2 antibodies treatment effectively reduced the level of MDSC in tumors, but no changes were observed for M1/M2 macrophages (online supplemental figure 9).

### HER2-DC1 i.t. and anti-HER2 antibodies combination treatment require FCγR

ADCC, by triggering FCγRIII on NK cells, is the key mechanism that therapeutic antibodies utilize to act against tumors.^5^ To examine whether tumor regression induced by HER2-DC1 i.t. in combination with anti-HER2 antibodies treatment also require ADCC activity, we used FcγR-deficient (FcγRI/III-KO) mice model. Interestingly, antitumor efficacy of the combination treatment with HER2-DC1 i.t. and anti-HER2 antibodies was abrogated and failed to control TUBO tumor growth in FcγR KO mice (figure 5E). This data strongly suggests that ADCC activity is important for antitumor efficacy of HER2-DC1 i.t. and anti-HER antibodies combination treatment in HER2^pos^ BC.

### Molecular changes after HER2-DC1 i.t. and anti-HER2 antibodies combination treatment

**Figure 6:**
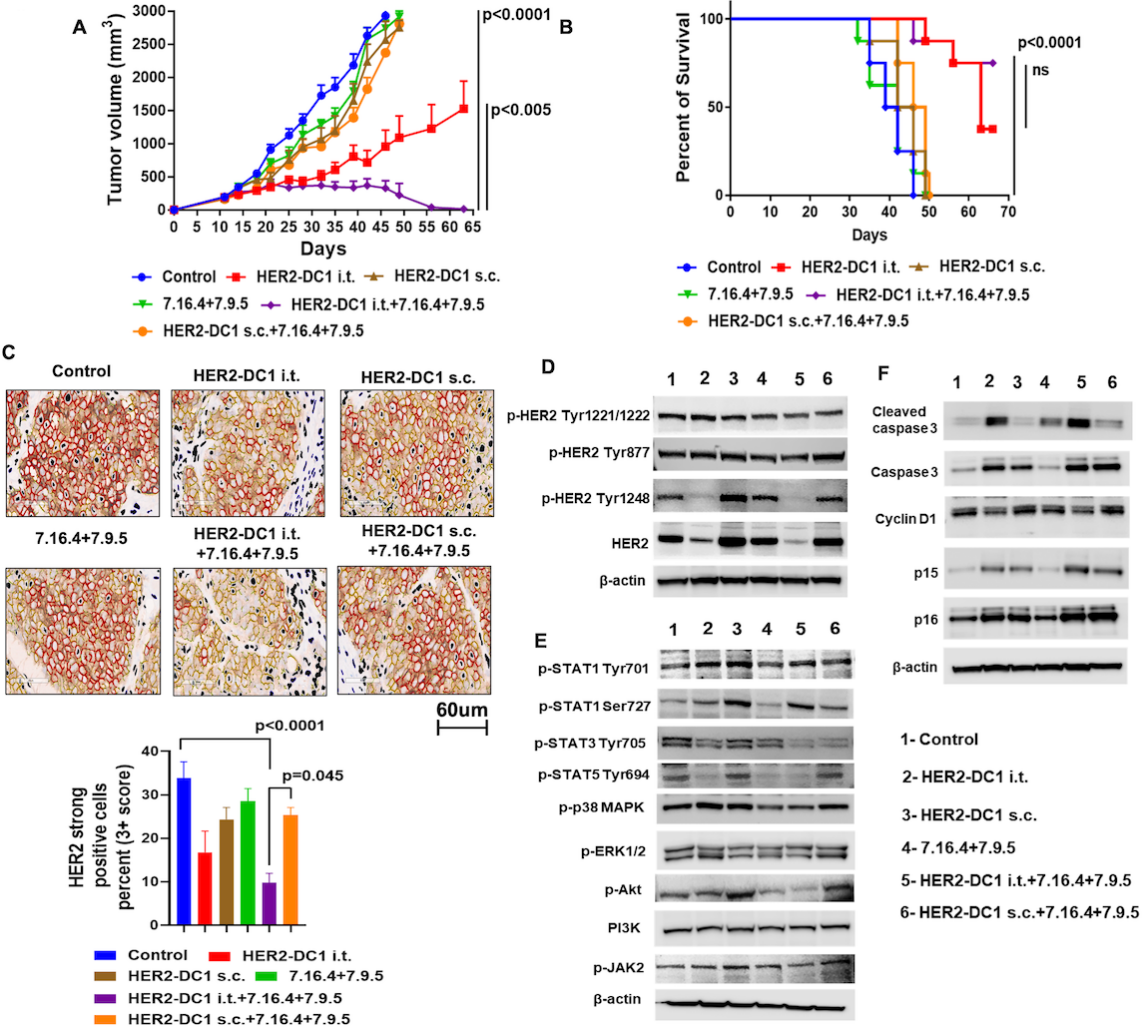
HER2-DC1 i.t. in combination with HER2 antibodies treatment induced molecular alterations in HER2^pos^ breast cancer model. (A) BALB/c mice bearing TUBO tumors were treated with HER2-DC1 i.t. alone, HER2-DC1 s.c. alone, anti-HER2 antibodies alone (both clones 7.16.4+7.9.5), HER2-DC1 i.t. in combination with anti-HER2 antibodies (both clones 7.16.4+7.9.5), HER2-DC1 s.c. in combination with anti-HER2 antibodies (both clones 7.16.4+7.9.5) as described in methods. Tumor growth was monitored two times a week (n=8). (B) Survival curve (n=8). Mean±SEM. HER2-DC1 i.t. versus HER2-DC1 i.t.+7.16.4+7.9.5 (p<0.005) in (A) and (ns) in (B), other groups versus HER2-DC1 i.t.+7.16.4+7.9.5 (p<0.0001) in (A) and (B). (C) Cell surface expression of HER2 in the tumors of experimental mice was analyzed by immunohistochemistry. (D–F) Protein expression of HER2, p-HER2 Tyr1248, p-HER2 Tyr877, p-HER2 Tyr1221/1222, p-Akt, PI3K, p-JAK2, p-ERK1/2, p-P38 MAPK, p-STAT1 Tyr701, p-STAT1 Ser727, p-STAT3 Tyr705, p-STAT5 Tyr694, cyclin D1, caspase 3, cleaved caspase 3, p15 and p16 in the tumors of experimental mice was examined by Western blot. Results are shown as mean±SEM of at least three independent experiments. HER2, human epidermal growth factor receptor 2; HER2-DC1, HER2 peptide-pulsed type 1 polarized dendritic cell; i.t., intratumoral; ns, not significant; s.c., subcutaneous.

We examined molecular changes mediated after HER2-DC1 i.t. combined with anti-HER2 antibodies treatment in the HER2^pos^ TUBO tumor model. As expected, HER2-DC1 i.t. in combination with anti-HER2 antibodies enhanced antitumor effects with tumor regression in 80% of mice (figure 6A, B). Importantly, we observed a significant decrease in the level HER2 surface expression in the tumors of HER2-DC1 i.t. and anti-HER2 antibodies combination treatment group compared with HER2-DC1 s.c. and anti-HER2 antibodies combination treatment (figure 6C). In addition, HER2-DC1 i.t. and anti-HER2 antibodies combination treatment reduced expression and autophosphorylation of HER2 (HER2, p-HER2 Tyr1248, p-HER2 Tyr877 and p-HER2 Tyr1221/1222) in tumors compared with HER2-DC1 s.c. and anti-HER2 antibodies combination treatment or monotherapy (figure 6D). Next, HER2-DC1 i.t. and anti-HER2 antibodies combination treatment induced STAT1 activation (p-STAT1 Tyr701 and p-STAT1 Ser727) and reduced activation of various other intracellular signaling molecules such as p-STAT3, p-STAT5, p-p38MAPK, p-ERK1/2, p-Akt and p-JAK2 in tumors (figure 6E).

To further test the senescence inducing potential of HER2-DC1 i.t. and anti-HER2 antibodies combination treatment, expression of senescence marker proteins p15 and p16 was examined. Tumors from HER2-DC1 i.t. combined with anti-HER2 antibodies treatment group had increased expression of p15 and p16 proteins compared with HER2-DC1 s.c. combined with anti-HER2 antibodies or monotherapy (figure 6F). The HER2 signaling pathway directly regulates cyclin D1 expression, resulting in cell cycle regulation and cancer cell proliferation.^34^ As shown in figure 6F, cyclin D1 expression was downregulated after HER2-DC1 i.t. and anti-HER2 antibodies combination treatment. Furthermore, increased expression of apoptosis markers caspase 3 and cleaved caspase 3 in tumors, suggests the additive effect of HER2-DC1 i.t. combined with anti-HER2 antibodies treatment in mediating HER2^pos^ tumor regression (figure 6F). Taken together, these data suggest that HER2-DC1 i.t. and anti-HER2 antibodies combination treatment regulates HER2 oncogenic singling pathways.

### HER2-DC1 i.t. in combination with anti-HER2 antibodies is more effective than standard chemotherapy paclitaxel with anti-HER2 antibodies treatment

**Figure 7:**
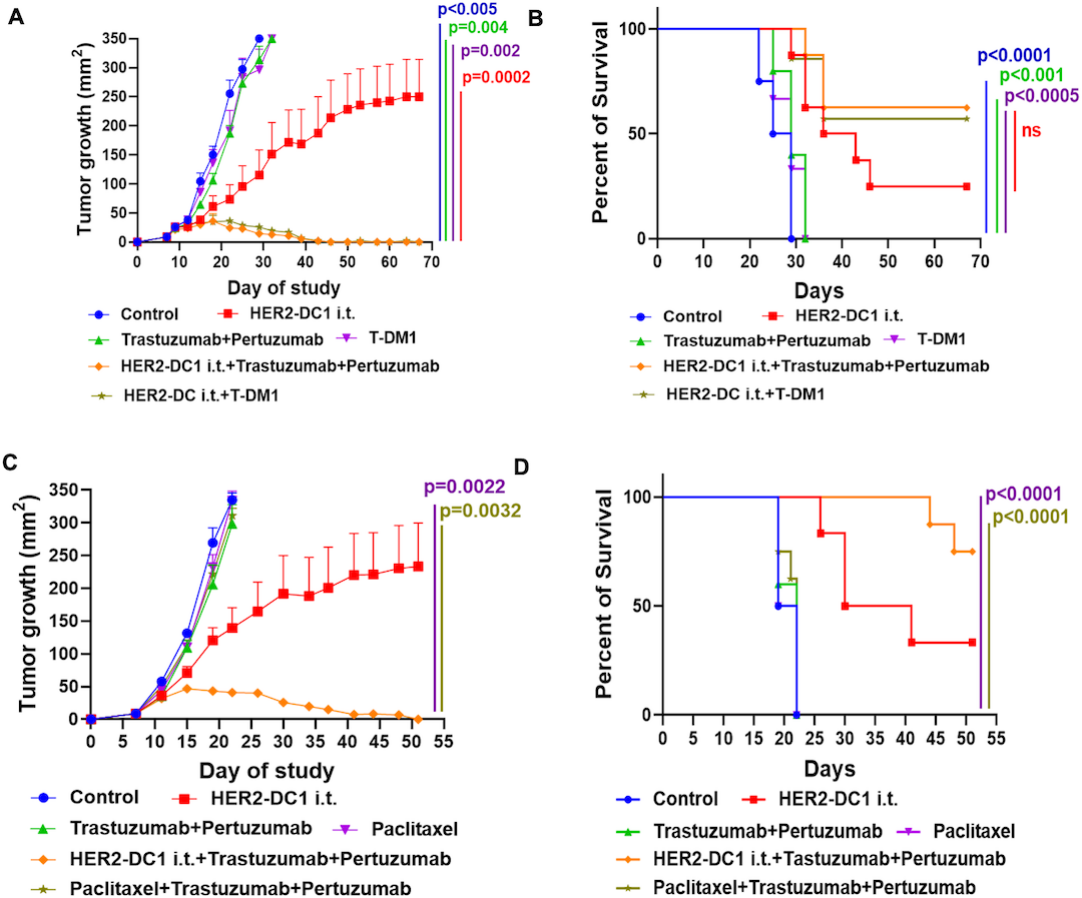
HER2-DC1 i.t. combined with anti-HER2 antibodies treatment is more effective compared with standard chemotherapy in combination with anti-HER2 antibodies. (A) Tumor growth curves and (B) survival curves of CT26 hHER2 tumor bearing mice received various treatments as indicated (n=8). Mean±SEM. Control versus HER2-DC1 i.t.+trastuzumab+pertuzumab and HER2-DC1 i.t. +T-DM1 (p<0.005) in (A) and (p<0.0001) in (B). HER2-DC1 i.t. versus HER2-DC1 i.t. +trastuzumab+pertuzumab and HER2-DC1 i.t. +T-DM1 (p=0.0002) in (A) and (ns) in (B). Trastuzumab +pertuzumab versus HER2-DC1 i.t. +trastuzumab+pertuzumab and HER2-DC1 i.t. +T-DM1 (p=0.004) in (A) and (p<0.001) in (B). T-DM1 versus HER2-DC1 i.t. +trastuzumab+pertuzumab and HER2-DC1 i.t. +T-DM1 (p=0.002) in (B) and (p<0.0005) in (B). (C) Tumor growth curves and (D) survival curves in CT26 hHER2 bearing tumors in different treatment conditions as indicated (n=6–8). Paclitaxel versus HER2-DC1 i.t. +trastuzumab+pertuzumab (p=0.0022) in (C) and (p<0.0001) in (D). Paclitaxel +trastuzumab+pertuzumab versus HER2-DC1 i.t. +trastuzumab+pertuzumab (p=0.0032) in (C) and (p<0.0001) in (D). HER2, human epidermal growth factor receptor 2; HER2-DC1, HER2 peptide-pulsed type 1 polarized dendritic cell; i.t., intratumoral; ns, not significant; T-DM1, trastuzumab emtansine.

To validate the clinical relevance of HER2-DC1 i.t. treatment in combination with anti-HER2 antibodies, we used the CT26 hHER2 tumor model. As shown in figure 7A, B, HER2-DC1 i.t. combined with trastuzumab and pertuzumab treatment showed a more remarkable antitumor response with tumor regression in 65% of treated mice and prolonged survival. The combination of HER2-DC1 i.t. and T-DM1 also significantly inhibited CT26 hHER2 tumor growth in 60% of treated mice and improved survival (Figure 7A, B). These mice with tumor regression also rejected secondary CT26 hHER2 tumor challenge and TUBO tumor challenge, thus highlighting the presence of enduring antitumor immune response (figure 7A). We further compared the antitumor efficacy of HER2-DC1 i.t. in combination with anti-HER2 antibodies treatment versus current standard chemotherapy paclitaxel combined with anti-HER2 antibodies in the CT26 hHER2 tumor model. Paclitaxel treatment alone was not able to delay the tumor growth in the CT26 hHER2 tumor model (figure 7C). A similar effect was also observed in the TUBO tumor model for paclitaxel treatment (online supplemental figure 10). Enhanced antitumor reactivity with tumor regression in 75% of treated mice was observed for the HER2-DC1 i.t. combined with anti-HER2 antibodies trastuzumab and pertuzumab treatment when compared with paclitaxel combined with anti-HER2 antibodies trastuzumab and pertuzumab (Figure 7C, D). Taken together, these results suggest that HER2-DC1 i.t. is more effective when combined with anti-HER2 antibodies compared with paclitaxel in combination with anti-HER2 antibodies in HER2^pos^ BC.

## Discussion

HER2 targeted therapies trastuzumab and pertuzumab in combination with chemotherapy in the neoadjuvant setting is effective in a subset of patients with HER2^pos^ BC, but in parallel hematologic and non-hematologic toxicities have been frequently noted in a substantial fraction of patients.^13 14^ This suggests that an alternate combination treatment approach is needed to enhance the clinical response and overcome chemotherapy induced toxicities in patients with HER2^pos^ BC. This present study describes the antitumor efficacy of HER2-DC1 i.t. combined with anti-HER2 antibodies treatment in three different HER2^pos^ BC models such as the rat HER2 expressing TUBO tumor model, BALB-HER2 transgenic spontaneous tumor model and clinically relevant human HER2 expressing CT26 tumor model. BALB-HER2 transgenic model express rat HER2 and represents human HER2^pos^ BC, which spontaneously develop pre-invasive mammary lesions and progresses to invasive tumors and metastasis.^35^ The TUBO cells were cloned from a BALB-HER2 transgenic mouse mammary tumors and grew progressively in the mammary gland of wild type BALB/c mice.^36^ These two models were used to test the antitumor efficacy of HER2-DC1 i.t. combined with anti-rat HER2 antibodies 7.16.4 and 7.9.5 that mimic trastuzumab and pertuzumab, respectively. The CT26 hHER2 was established by engineering CT-26 cells to express human HER2^37^ and this tumor model was used to compare the therapeutic efficacy of HER2-DC1 i.t. combined with anti-human HER2 antibodies trastuzumab and pertuzumab or T-DM1 versus chemotherapy paclitaxel combined with trastuzumab and pertuzumab.

Our study shows that HER2-DC1 i.t. combined with anti-HER2 antibodies treatment is more effective than standard chemotherapy paclitaxel combined with anti-HER2 antibodies and induces complete tumor regression in the HER2^pos^ BC model. The superior antitumor response following HER2-DC1 i.t. in combination with anti-HER2 antibodies involves tumor infiltration of CD4^+^ and CD8^+^ T cells, B cells, NKT cells and NK cells. Although HER2-DC1 s.c. combined with anti-HER2 antibodies treatment reduced tumor burden and improved survival, it was not able to prolong infiltration of immune cells into the tumor and completely arrest tumor growth in a HER2^pos^ BC model. Generation of a strong anti-HER2 Th1 immune response in the tumor draining lymph node, spleen and peripheral blood are critical for the inhibitory effect of the combination treatment with HER2-DC1 i.t. and anti-HER2 antibodies on tumor growth, which was supported by detection of increased Th1 cytokines IFN-γ and TNF-α. However, HER2-DC1 s.c. combined with anti-HER2 antibodies treatment failed to induce enhanced anti-HER2 Th1 immune response in the tumor draining lymph node and peripheral blood, which further supports the inability of generating strong and sustained antitumor immunity for this combination treatment approach in a HER2^pos^ BC model. IFN-γ secreted by tumor-reactive CD4^+^ Th1 cells and CD8^+^ cytotoxic T cells exhibit pleiotropic effects during the anti-tumor immune response. The pleiotropic effects of IFN-γ include cell-specific regulation of inflammatory signaling pathways, pro-apoptosis, and cancer cell proliferation arrest.^38^ In patients with HER2^pos^ BC, sustained secretion of IFN-γ was positively associated with response to the treatment and survival.^39 40^ Our clinical finding supports this study that restoration of anti-HER2 Th1 immunity using intralesional or intranodal HER2-DC1 vaccine was able to improve the pCR in patients with HER2^pos^ BC.^41^

Predominance of CD4^+^ and CD8^+^ T cell infiltration into the tumor bed is a positive predictive marker for the outcome of targeted therapies and is associated with a favorable prognosis in patients with BC. Moreover, higher levels of tumor-infiltrating lymphocytes are considered beneficial for the efficacy of trastuzumab treatment in patients with HER2^pos^ BC.^42 43^ The tumor reactive CD4^+^ Th1 cells can enforce activation of CD8^+^ T cells to further potentiate the treatment benefits and improve patient with BC survival.^44^ Very recently it was observed that cDC1 primarily drives tumor antigen specific CD4^+^T cells activation and help the priming and infiltration of cytotoxic CD8^+^ T cells into the tumors.^45^ The cDC1 can process and present tumor antigen derived immunogenic peptides to CD4^+^ T cells via MHC class II/T cell antigen receptor engagement and trigger antitumor specific CD4^+^Th1 cells activation in immunologic tumors and solid tumors.^44^ Our recent findings strongly support the therapeutic benefits of using multiepitope MHC class II tumor antigenic peptides pulsed DC1 therapy in driving CD4^+^ Th1 immunity in BC subtypes.^41 46^ Notably, pulsing with MHC class II HER2 peptides p5, p435 and p1209 has been identified to drive anti-HER2 CD4^+^ Th1 immune response in HER2^pos^ BC.^29 41^ Previous studies have demonstrated the major advantages of tumor antigen-pulsed DC i.t. delivery in enhancing the efficacy of targeted agents and increasing CD4^+^ and CD8^+^ T cell tumor infiltration in patients with advanced stage solid tumors.^30 31^ Recently, treatment with DC i.t. combined with local radiotherapy has been shown to increase tumor antigen specific CD8^+^ T cell infiltration in poorly immunogenic preclinical tumor models.^32^ In this study, high tumor infiltration of CD4^+^ and CD8^+^ T cells was observed for HER2-DC1 i.t. combined with anti-HER2 antibodies treatment in the HER2^pos^ BC model. In contrast, synergistic effects of HER2-DC1 i.t. and anti-HER2 antibodies were lost when CD4^+^ or CD8^+^ T cells were depleted. This data demonstrates the critical role for CD4^+^ and CD8^+^ T cells in HER2-DC1 i.t. plus anti-HER2 antibodies combination treatment for inducing an effective antitumor response in HER2^pos^ BC.

Adoptive immunological memory mediated by tumor reactive CD4^+^ and CD8^+^ T cells play a key role in protective immunity to tumor antigens. Activated tumor reactive CD4^+^ and CD8^+^ T cells can give rise to effector and memory cells. Memory CD4^+^ and CD8^+^ T cells are differentiated into effector memory and central memory subsets. The effector CD4^+^ and CD8^+^ T cells may become terminally differentiated and show short-term antitumor effects.^47^ In contrast, effector memory/central memory CD4^+^ and CD8^+^ T cells can provide robust and enduring immunological protection against tumors.^47^ In the present study, increased accumulation of CD4^+^ effector memory, central memory and effector T cells was observed in the TME following treatment with HER2-DC1 i.t. in combination with anti-HER2 antibodies. Enhancement of CD8^+^ central memory T cells was also noted for HER2-DC1 i.t. and anti-HER2 antibodies combination treatment. In this study, enduring antitumor immune response is identified as a key therapeutic feature for HER2-DC1 i.t. combined with anti-HER2 antibodies treatment, supported by rejection of secondary HER2^pos^ tumor challenge and detection of increased levels of both immune stimulatory cytokines and pro-inflammatory chemokines. Previous clinical trials revealed very promising efficacy for combination treatment of IL-12 with trastuzumab with sustained production of immune stimulatory cytokines and IFN-γ in patients with metastatic BC.^39^ Immune stimulatory cytokine and chemokine production following immunotherapy contributes to improved tumor antigen priming, driving more effector immune cells including CD4, CD8, NK, NKT and B cells in the TME, and enhancing cytolytic activity.^48 49^ Our findings suggest that HER2-DC1 i.t. combined with anti-HER2 antibodies treatment also drives high infiltration of NK cells, NKT cells and B cells to further enhance the antitumor immune response in the HER2^pos^ BC model.

Evidence has shown that HER2 targeted antibodies induce ADCC by triggering FCγRIII on NK cells and act against HER2^pos^ disease.^5^ A clinical trial showed that trastuzumab induced ADCC with pCR in patients with HER2^pos^ BC. However, loss of ADCC activity for trastuzumab treatment was also noted in patients with metastatic HER2^pos^ BC.^50^ DC vaccine therapy in patients with HER2^pos^ metastatic BC elicited production of specific anti-HER2 antibodies and CD4 Th1 immunity resulting in complete response.^51^ Our study identified that combination treatment with HER2-DC1 i.t. and anti-HER2 antibodies induces ADCC for the enhanced antitumor response, supported by the failure to block tumor aggression and to induce complete tumor growth arrest in a FcγR KO model. In the HER2^pos^ BC model, high tumor infiltration of NK cells was also noted following HER2-DC1 i.t. and anti-HER2 antibodies combination treatment. Taken together, this study provides evidence that HER2-DC1 i.t. and anti-HER2 antibodies combination treatment also induced ADCC for tumor regression in HER2^pos^ BC.

Other investigators have shown that a DC i.t. treatment approach in combination with local radiotherapy can increase the migration capacity of i.t. injected DC as well as control the growth of treated primary and untreated distant tumors by mediating the frequency of circulatory tumor antigen specific CD8^+^ T cells.^32^ We observed generation of systemic antitumor immunity following combined treatment with HER2-DC i.t. and anti-HER2 antibodies in the HER2^pos^ BC model used, which was evidenced by attenuated growth of untreated distant tumors. Notably, our study identified an increased frequency of HER2-DC1 in the treated primary tumors and their migration, with prolonged survival, into the untreated distant tumors following HER2-DC i.t. and anti-HER2 antibodies combination treatment in a HER2^pos^ BC model. Activation of tumor residing cDC1 in combination with radiotherapy has been shown to be critical to overcome acquired resistance to anti-PD-L1 therapy and enhance i.t. T cell infiltration in poor T cell infiltrating tumor models.^52^ An in-situ vaccination (ISV) strategy that activates tumor residing DCs in combination with radiotherapy was reported to induce regression of primary and untreated distant tumors in patients with lymphoma.^53^ In addition, i.t. administration of ISV was more effective in recruiting various DC subsets to treated primary and untreated distant tumors compared with s.c. administration, leading to enhanced tumor-associated antigen cross presentation, T cell priming and strong systemic antitumor immunity.^53^ Our study identified the enrichment of cDC1 and MoDCs in treated primary tumors and cDC2 in both treated primary and untreated distant tumors after HER2-DC1 i.t. in combination with anti-HER2 antibodies treatment. The increased frequency of cDC2 in untreated distant tumors may drive CD4^+^ T cells and B cells to control the distant tumor growth. Further studies are required to better understand how HER2-DC1 i.t. in combination with anti-HER2 antibodies treatment induces cDC2 enrichment and contributes to tumor growth arrest in untreated distant tumors in HER2^pos^ BC.

Overexpression and constitutive activation/autophosphorylation of HER2 is associated with more aggressive disease and poor prognosis in patients with HER2^pos^ BC.^1^ The activated HER2 protein recruits and regulates various intracellular signaling proteins PI3K/Akt, JAK2, STAT3 and STAT5 to induce cancer cell proliferation, differentiation, and survival in HER2^pos^ BC.^54–56^ Trastuzumab treatment has been shown to block HER2-mediated PI3K/Akt signaling activation in HER2^pos^ BC cells. However, HER2 dependent blockade of PI3K/Akt proteins can lead to compensatory activation of the MAPK/ERK signaling pathway.^57^ Importantly, various studies have shown limited inhibitory activity of trastuzumab on HER2 activation/autophosphorylation in HER2^pos^ BC.^58^ Our study provides molecular evidence that HER2-DC1 i.t. and anti-HER2 antibodies combination treatment remarkably inhibited expression and activation/autophosphorylation of HER2, resulting in diminished activation of HER2-mediated signaling proteins Akt, JAK2, STAT3, STAT5, MAPK and ERK in the HER2^pos^ BC model. IFN-γ has the potential to mediate degradation of HER2 through a ubiquitin proteasomal pathway in HER2^pos^ BC cells.^25^ IFN-γ in combination with anti-HER2 antibodies, trastuzumab and pertuzumab, has been shown to increase senescence and apoptosis via STAT1 signaling activation in HER2^pos^ BC cells.^28^ This study provides confirmative evidence for the previous finding that enhanced STAT1 activation was observed following HER2-DC1 i.t. combined with anti-HER2 antibodies treatment in a HER2^pos^ BC model. The HER2 signaling pathway can directly regulate cyclin D1 expression resulting in cell cycle progression and cancer cell proliferation.^34^ It has been reported that HER2^pos^ BC cells can escape from HER2-targeted agents trastuzumab, pertuzumab and T-DM1 and acquire defects in the mechanism of apoptosis.^59 60^ In the present study of HER2^pos^ BC model, enhanced apoptosis, senescence, and inhibition of cell cycle progression appear to be a beneficial effect of HER2-DC1 i.t. combined with anti-HER2 antibodies treatment, which was supported by increased caspase 3, cleaved-caspase 3, p15 and p16 expression, and reduced expression of cyclin D1.

In summary, we propose that HER2-DC1 i.t. in combination with anti-HER2 antibodies is an effective therapeutic strategy to target HER2-mediated signaling pathways in HER2^pos^ BC. Generation of systemic and enduring antitumor immunity and greater inhibition of primary and distant tumors in HER2^pos^ BC can be accomplished with combining HER2-DC1 i.t. and anti-HER2 antibodies. Our data suggest that HER2-DC1 i.t. combined with anti-HER2 antibodies can be tested as an effective alternative therapeutic strategy to standard chemotherapy combined with anti-HER2 antibodies in HER2^pos^ BC, and indeed supports the general notion that targeted, lower toxicity agents can be combined with DC-based immunotherapy to improve therapeutic outcomes. A clinical trial is ongoing to address the feasibility of HER2-DC1 i.t. plus anti-HER2 antibodies in the neoadjuvant setting for patients with HER2^pos^ BC (ClinicalTrials.gov: NCT03387553).

## Data Availability

Data are available upon reasonable request. All data relevant to the study are included in the article or uploaded as supplementary information.

## References

[1] Loibl S, Gianni L. Her2-Positive breast cancer. Lancet 2017;389:2415–29. 10.1016/S0140-6736(16)32417-5 27939064

[2] Swain SM, Miles D, Kim S-B, et al. Pertuzumab, trastuzumab, and docetaxel for HER2-positive metastatic breast cancer (CLEOPATRA): end-of-study results from a double-blind, randomised, placebo-controlled, phase 3 study. Lancet Oncol 2020;21:519–30. 10.1016/S1470-2045(19)30863-0 32171426

[3] von Minckwitz G, Procter M, de Azambuja E, et al. Adjuvant pertuzumab and trastuzumab in early HER2-positive breast cancer. N Engl J Med 2017;377:122–31. 10.1056/NEJMoa1703643 28581356 PMC5538020

[4] Griguolo G, Pascual T, Dieci MV, et al. Interaction of host immunity with HER2-targeted treatment and tumor heterogeneity in HER2-positive breast cancer. J Immunother Cancer 2019;7:90. 10.1186/s40425-019-0548-6 30922362 PMC6439986

[5] Tóth G, Szöőr Árpád, Simon L, et al. The combination of trastuzumab and pertuzumab administered at Approved doses may delay development of trastuzumab resistance by additively enhancing antibody-dependent cell-mediated cytotoxicity. MAbs 2016;8:1361–70. 10.1080/19420862.2016.1204503 27380003 PMC5058622

[6] Honkanen TJ, Moilanen T, Karihtala P, et al. Prognostic and predictive role of spatially positioned tumour infiltrating lymphocytes in metastatic HER2 positive breast cancer treated with trastuzumab. Sci Rep 2017;7:18027. 10.1038/s41598-017-18266-1 29269742 PMC5740084

[7] Varadan V, Gilmore H, Miskimen KLS, et al. Immune signatures following single dose trastuzumab predict pathologic response to PreoperativeTrastuzumab and chemotherapy in HER2-positive early breast cancer. Clin Cancer Res 2016;22:3249–59. 10.1158/1078-0432.CCR-15-2021 26842237 PMC5439498

[8] Kono K, Sato E, Naganuma H, et al. Trastuzumab (Herceptin) enhances class I-restricted antigen presentation recognized by HER-2/neu-specific T cytotoxic lymphocytes. Clin Cancer Res 2004;10:2538–44. 10.1158/1078-0432.CCR-03-0424 15073134

[9] Gall VA, Philips AV, Qiao N, et al. Trastuzumab increases HER2 uptake and cross-presentation by dendritic cells. Cancer Res 2017;77:5374–83. 10.1158/0008-5472.CAN-16-2774 28819024 PMC5626640

[10] Gianni L, Pienkowski T, Im Y-H, et al. 5-Year analysis of neoadjuvant pertuzumab and trastuzumab in patients with locally advanced, inflammatory, or early-stage HER2-positive breast cancer (NeoSphere): a multicentre, open-label, phase 2 randomised trial. Lancet Oncol 2016;17:791–800. 10.1016/S1470-2045(16)00163-7 27179402

[11] Early Breast Cancer Trialists’ Collaborative group (EBCTCG). Trastuzumab for early-stage, HER2-positive breast cancer: a meta-analysis of 13 864 women in seven randomised trials. Lancet Oncol 2021;22:1139–50. 10.1016/S1470-2045(21)00288-6 34339645 PMC8324484

[12] Schneeweiss A, Chia S, Hickish T, et al. Pertuzumab plus trastuzumab in combination with standard neoadjuvant anthracycline-containing and anthracycline-free chemotherapy regimens in patients with HER2-positive early breast cancer: a randomized phase II cardiac safety study (TRYPHAENA). Ann Oncol 2013;24:2278–84. 10.1093/annonc/mdt182 23704196

[13] Dang C, Guo H, Najita J, et al. Cardiac outcomes of patients receiving adjuvant Weekly paclitaxel and trastuzumab for node-negative, ErbB2-positive breast cancer. JAMA Oncol 2016;2:29–36. 10.1001/jamaoncol.2015.3709 26539793 PMC5654518

[14] Tolaney SM, Barry WT, Dang CT, et al. Adjuvant paclitaxel and trastuzumab for node-negative, HER2-positive breast cancer. N Engl J Med 2015;372:134–41. 10.1056/NEJMoa1406281 25564897 PMC4313867

[15] von Minckwitz G, Huang C-S, Mano MS, et al. Trastuzumab emtansine for residual invasive HER2-positive breast cancer. N Engl J Med 2019;380:617–28. 10.1056/NEJMoa1814017 30516102

[16] Hunter FW, Barker HR, Lipert B, et al. Mechanisms of resistance to trastuzumab emtansine (T-DM1) in HER2-positive breast cancer. Br J Cancer 2020;122:603–12. 10.1038/s41416-019-0635-y 31839676 PMC7054312

[17] Tolaney SM, Tayob N, Dang C, et al. Adjuvant trastuzumab emtansine versus paclitaxel in combination with trastuzumab for stage I HER2-positive breast cancer (ATEMPT): a randomized clinical trial. J Clin Oncol 2021;39:2375–85. 10.1200/JCO.20.03398 34077270

[18] Modi S, Saura C, Yamashita T, et al. Trastuzumab Deruxtecan in previously treated HER2-positive breast cancer. N Engl J Med 2020;382:610–21. 10.1056/NEJMoa1914510 31825192 PMC7458671

[19] Esteva FJ, Hubbard-Lucey VM, Tang J, et al. Immunotherapy and targeted therapy combinations in metastatic breast cancer. Lancet Oncol 2019;20:e175–86. 10.1016/S1470-2045(19)30026-9 30842061

[20] Costa R, Zaman S, Sharpe S, et al. A brief report of toxicity end points of HER2 vaccines for the treatment of patients with HER2^+^ breast cancer. Drug Des Devel Ther 2019;13:309–16. 10.2147/DDDT.S188925 PMC6338114 30679903

[21] Griguolo G, Serna G, Pascual T, et al. Immune microenvironment characterisation and dynamics during anti-HER2-based neoadjuvant treatment in HER2-positive breast cancer. NPJ Precis Oncol 2021;5:23. 10.1038/s41698-021-00163-6 33742063 PMC7979716

[22] Wculek SK, Cueto FJ, Mujal AM, et al. Dendritic cells in cancer immunology and immunotherapy. Nat Rev Immunol 2020;20:7–24. 10.1038/s41577-019-0210-z 31467405

[23] Datta J, Rosemblit C, Berk E, et al. Progressive loss of anti-HER2 CD4^+^ T-helper type 1 response in breast tumorigenesis and the potential for immune restoration. Oncoimmunology 2015;4:e1022301. 10.1080/2162402X.2015.1022301 26451293 PMC4589053

[24] Datta J, Berk E, Xu S, et al. Anti-HER2 CD4(+) T-helper type 1 response is a novel immune correlate to pathologic response following neoadjuvant therapy in HER2-positive breast cancer. Breast Cancer Res 2015;17:71. 10.1186/s13058-015-0584-1 25997452 PMC4488128

[25] Jia Y, Kodumudi KN, Ramamoorthi G, et al. Th1 cytokine interferon gamma improves response in HER2 breast cancer by modulating the ubiquitin proteasomal pathway. Mol Ther 2021;29:1541–56. 10.1016/j.ymthe.2020.12.037 33412308 PMC8058490

[26] Namjoshi P, Showalter L, Czerniecki BJ, et al. T-Helper 1-type cytokines induce apoptosis and loss of HER-family oncodriver expression in murine and human breast cancer cells. Oncotarget 2019;10:6006–20. 10.18632/oncotarget.10298 31666931 PMC6800266

[27] Zhang S, Kohli K, Black RG, et al. Systemic interferon-γ increases MHC class I expression and T-cell infiltration in cold tumors: results of a phase 0 clinical trial. Cancer Immunol Res 2019;7:1237–43. 10.1158/2326-6066.CIR-18-0940 31171504 PMC6677581

[28] Rosemblit C, Datta J, Lowenfeld L, et al. Oncodriver inhibition and CD4^+^ Th1 cytokines cooperate through Stat1 activation to induce tumor senescence and apoptosis in HER2+ and triple negative breast cancer: implications for combining immune and targeted therapies. Oncotarget 2018;9:23058–77. 10.18632/oncotarget.25208 29796172 PMC5955413

[29] Kodumudi KN, Ramamoorthi G, Snyder C, et al. Sequential anti-PD1 therapy following dendritic cell vaccination improves survival in a HER2 mammary carcinoma model and identifies a critical role for CD4 T cells in mediating the response. Front Immunol 2019;10:1939. 10.3389/fimmu.2019.01939 31475002 PMC6702967

[30] Kolstad A, Kumari S, Walczak M, et al. Sequential intranodal immunotherapy induces antitumor immunity and correlated regression of disseminated follicular lymphoma. Blood 2015;125:82–9. 10.1182/blood-2014-07-592162 25293773

[31] Cox MC, Castiello L, Mattei M, et al. Clinical and antitumor immune responses in relapsed/refractory follicular lymphoma patients after intranodal injections of IFNα-Dendritic cells and rituximab: a phase I clinical trial. Clin Cancer Res 2019;25:5231–41. 10.1158/1078-0432.CCR-19-0709 31171545

[32] Oba T, Makino K, Kajihara R, et al. In situ delivery of iPSC-derived dendritic cells with local radiotherapy generates systemic antitumor immunity and potentiates PD-L1 blockade in preclinical poorly immunogenic tumor models. J Immunother Cancer 2021;9:e002432. 10.1136/jitc-2021-002432 34049930 PMC8166607

[33] Granato A, Hayashi EA, Baptista BJA, et al. Il-4 regulates Bim expression and promotes B cell maturation in synergy with BAFF conferring resistance to cell death at negative selection checkpoints. J Immunol 2014;192:5761–75. 10.4049/jimmunol.1300749 24835393

[34] Lee RJ, Albanese C, Fu M, et al. Cyclin D1 is required for transformation by activated neu and is induced through an E2F-dependent signaling pathway. Mol Cell Biol 2000;20:672–83. 10.1128/MCB.20.2.672-683.2000 10611246 PMC85165

[35] Hosseini H, Obradović MMS, Hoffmann M, et al. Early dissemination seeds metastasis in breast cancer. Nature 2016;540:552–8. 10.1038/nature20785 27974799 PMC5390864

[36] Sow HS, Benonisson H, Brouwers C, et al. Immunogenicity of rat-neu^+^ mouse mammary tumours determines the T cell-dependent therapeutic efficacy of anti-neu monoclonal antibody treatment. Sci Rep 2020;10:3933. 10.1038/s41598-020-60893-8 32127568 PMC7054273

[37] Parihar R, Dierksheide J, Hu Y, et al. Il-12 enhances the natural killer cell cytokine response to Ab-coated tumor cells. J Clin Invest 2002;110:983–92. 10.1172/JCI0215950 12370276 PMC151155

[38] Gocher AM, Workman CJ, Vignali DAA. Interferon-γ: teammate or opponent in the tumour microenvironment? Nat Rev Immunol 2022;22:158-172. 10.1038/s41577-021-00566-3 34155388 PMC8688586

[39] Parihar R, Nadella P, Lewis A, et al. A phase I study of interleukin 12 with trastuzumab in patients with human epidermal growth factor receptor-2-overexpressing malignancies: analysis of sustained interferon gamma production in a subset of patients. Clin Cancer Res 2004;10:5027–37. 10.1158/1078-0432.CCR-04-0265 15297404

[40] Mani A, Roda J, Young D, et al. A phase II trial of trastuzumab in combination with low-dose interleukin-2 (IL-2) in patients (PTS) with metastatic breast cancer (MBC) who have previously failed trastuzumab. Breast Cancer Res Treat 2009;117:83–9. 10.1007/s10549-008-0251-7 19051009 PMC2997435

[41] Lowenfeld L, Mick R, Datta J, et al. Dendritic Cell Vaccination Enhances Immune Responses and Induces Regression of HER2^pos^ DCIS Independent of Route: Results of Randomized Selection Design Trial. Clin Cancer Res 2017;23:2961–71. 10.1158/1078-0432.CCR-16-1924 27965306

[42] Solinas C, Ceppi M, Lambertini M, et al. Tumor-Infiltrating lymphocytes in patients with HER2-positive breast cancer treated with neoadjuvant chemotherapy plus trastuzumab, lapatinib or their combination: a meta-analysis of randomized controlled trials. Cancer Treat Rev 2017;57:8–15. 10.1016/j.ctrv.2017.04.005 28525810

[43] Luen SJ, Salgado R, Fox S, et al. Tumour-Infiltrating lymphocytes in advanced HER2-positive breast cancer treated with pertuzumab or placebo in addition to trastuzumab and docetaxel: a retrospective analysis of the CLEOPATRA study. Lancet Oncol 2017;18:52–62. 10.1016/S1470-2045(16)30631-3 27964843 PMC5477653

[44] Basu A, Ramamoorthi G, Albert G, et al. Differentiation and Regulation of T_H_ Cells: A Balancing Act for Cancer Immunotherapy. Front Immunol 2021;12:669474. 10.3389/fimmu.2021.669474 34012451 PMC8126720

[45] Ferris ST, Durai V, Wu R, et al. cDC1 prime and are licensed by CD4^+^ T cells to induce anti-tumour immunity. Nature 2020;584:624–9. 10.1038/s41586-020-2611-3 32788723 PMC7469755

[46] Basu A, Albert GK, Awshah S, et al. Identification of Immunogenic MHC Class II Human HER3 Peptides that Mediate Anti-HER3 CD4^+^ Th1 Responses and Potential Use as a Cancer Vaccine. Cancer Immunol Res 2022;10:108–25. 10.1158/2326-6066.CIR-21-0454 34785506 PMC9414303

[47] Seder RA, Ahmed R. Similarities and differences in CD4+ and CD8+ effector and memory T cell generation. Nat Immunol 2003;4:835–42. 10.1038/ni969 12942084

[48] Berraondo P, Sanmamed MF, Ochoa MC, et al. Cytokines in clinical cancer immunotherapy. Br J Cancer 2019;120:6–15. 10.1038/s41416-018-0328-y 30413827 PMC6325155

[49] Kohli K, Pillarisetty VG, Kim TS. Key chemokines direct migration of immune cells in solid tumors. Cancer Gene Ther 2022;29:10-21. 10.1038/s41417-021-00303-x 33603130 PMC8761573

[50] Petricevic B, Laengle J, Singer J, et al. Trastuzumab mediates antibody-dependent cell-mediated cytotoxicity and phagocytosis to the same extent in both adjuvant and metastatic HER2/neu breast cancer patients. J Transl Med 2013;11:307. 10.1186/1479-5876-11-307 24330813 PMC4029549

[51] Berzofsky JA, Terabe M, Trepel JB, et al. Cancer vaccine strategies: translation from mice to human clinical trials. Cancer Immunol Immunother 2018;67:1863–9. 10.1007/s00262-017-2084-x 29143114 PMC6759211

[52] Oba T, Long MD, Keler T, et al. Overcoming primary and acquired resistance to anti-PD-L1 therapy by induction and activation of tumor-residing cDC1s. Nat Commun 2020;11:5415. 10.1038/s41467-020-19192-z 33110069 PMC7592056

[53] Hammerich L, Marron TU, Upadhyay R, et al. Systemic clinical tumor regressions and potentiation of PD1 blockade with in situ vaccination. Nat Med 2019;25:814–24. 10.1038/s41591-019-0410-x 30962585

[54] Ruiz-Saenz A, Dreyer C, Campbell MR, et al. Her2 amplification in tumors activates PI3K/Akt signaling independent of HER3. Cancer Res 2018;78:canres.0430.2018–58. 10.1158/0008-5472.CAN-18-0430 PMC6779043 29760043

[55] Marotta LLC, Almendro V, Marusyk A, et al. The JAK2/STAT3 signaling pathway is required for growth of CD44⁺CD24⁻ stem cell-like breast cancer cells in human tumors. J Clin Invest 2011;121:2723–35. 10.1172/JCI44745 21633165 PMC3223826

[56] Britschgi A, Andraos R, Brinkhaus H, et al. Jak2/Stat5 inhibition circumvents resistance to PI3K/mTOR blockade: a rationale for cotargeting these pathways in metastatic breast cancer. Cancer Cell 2012;22:796–811. 10.1016/j.ccr.2012.10.023 23238015

[57] Serra V, Scaltriti M, Prudkin L, et al. Pi3K inhibition results in enhanced her signaling and acquired ERK dependency in HER2-overexpressing breast cancer. Oncogene 2011;30:2547–57. 10.1038/onc.2010.626 21278786 PMC3107390

[58] Pohlmann PR, Mayer IA, Mernaugh R. Resistance to trastuzumab in breast cancer. Clin Cancer Res 2009;15:7479–91. 10.1158/1078-0432.CCR-09-0636 20008848 PMC3471537

[59] Moody SE, Schinzel AC, Singh S, et al. PRKACA mediates resistance to HER2-targeted therapy in breast cancer cells and restores anti-apoptotic signaling. Oncogene 2015;34:2061–71. 10.1038/onc.2014.153 24909179 PMC4261061

[60] Carpenter RL, Lo H-W. Regulation of apoptosis by HER2 in breast cancer. J Carcinog Mutagen 2013;2013:003. 10.4172/2157-2518.S7-003 PMC4830426 27088047

