# Supplementary material for: Intratumoral delivery of dendritic cells plus anti-HER2 therapy triggers both robust systemic antitumor immunity and complete regression in HER2 mammary carcinoma
Source: J Immunother Cancer. 2022 Jun 16;10(6):e004841. doi: 10.1136/jitc-2022-004841 (PMC9204433; doi:10.1136/jitc-2022-004841)
Supplement: Supplementary data [file jitc-2022-004841supp001.pdf]

Supplemental materials

Intratumoral delivery of dendritic cells plus anti-HER2 therapy triggers both robust systemic antitumor immunity and complete regression in HER2 mammary carcinoma

Ganesan Ramamoorthi[1], Krithika N. Kodumudi[1], Colin Snyder[1], Payal Grover[3], Hongtao Zhang[3], Mark I. Greene[3], Amrita Basu[1], Corey Gallen[1], Doris Wiener[1], Ricardo L.B. Costa[2], Hyo S. Han[2], Gary Koski[4], Brian J. Czerniecki[1,2]

Authors affiliations

1 Clinical Science & Immunology Program, H. Lee Moffitt Cancer Center & Research Institute, Tampa, Florida, USA

2 Department of Breast Oncology, H. Lee Moffitt Cancer Center & Research Institute, Tampa, Florida, USA

3 Department of Pathology and Laboratory Medicine, Perelman Medical School, University of Pennsylvania, Philadelphia, Pennsylvania, USA

4 Department of Biological Sciences, Kent State University, Kent, Ohio, USA

Corresponding Author: Brian J. Czerniecki, Department of Breast Oncology, H. Lee Moffitt Cancer Center & Research Institute, Tampa, Florida, USA

Email: Brian.Czerniecki@moffitt.org

METHODS

Antibodies and reagents

Anti-HER2/neu antibodies 7.16.4 and 7.9.5 that mimic trastuzumab and pertuzumab, respectively, were a gift from Dr. Mark I. Greene (University of Pennsylvania). Anti-HER2/neu antibody clone 7.16.4 (Cat. No. BE0277) was also purchased from BioXCell, West Lebanon, NH. Trastuzumab, pertuzumab, T-DM1 and paclitaxel were obtained from the Infusion Center at H. Lee Moffitt Cancer Center and Research Institute. Anti-mouse CD4 (clone GK1.5, Cat. No. BP0003-1), anti-mouse CD8α (clone 2.43, Cat. No. BE0061) and InVivoMAb rat IgG2b isotype (clone LTF-2, Cat. No. BP0090) were purchased from BioXCell (West Lebanon, NH).

Cell line and culture condition

The TUBO cell line (kindly provided by Dr. Wei Zen Wei, Wayne State University) was derived from a spontaneous mammary carcinoma in BALB-HER2/neu transgenic mice. CT26 expressing human HER2/neu (CT26 hHER2) cell line was a gift from Dr. William E. Carson (The Ohio State University). Cells were cultured in RPMI 1640 medium (Cat. No. MT-10-040-CM, Corning, Corning, NY) supplemented with 10% heat-inactivated FBS (Cat. No. MT35010CV, Fisher Scientific), 1 mM sodium pyruvate (Cat. No. MT-25-000-C1, Corning), 2 mM L-glutamine (Cat. No. 25005CI, Fisher Scientific), 0.1 mM nonessential amino acids (Cat. No. 25-025-CI, Corning), 100 U/mL penicillin and 100 mg/ml streptomycin (Cat. No. MT-30-002-CI, Corning), 50 mg/mL gentamycin (Cat. No. 15750-060, Gibco), 0.5 mg/mL fungizone (Cat. No. 15290018, Gibco), and 0.05 mM 2-mercaptoethanol (Cat. No. 21985-023, Invitrogen). Cells were cultured in a humidified

incubator with 5% CO_2 at 37°C. Cell lines were tested and confirmed negative for mycoplasma (PlasmoTest, Cat. No. rep-pt1, Invitrogen, San Diego, CA) prior to the start of the experiments.

Mouse models

Wild-type female BALB/c mice and C57BL/6 mice at 6-8 weeks of age were purchased from Charles River Laboratories. FcRγ-deficient C.129P2(B6)-*Fcer1g*^{tm1Rav} N12 female mice were purchased from Taconic Biosciences and used at the age of 6-8 weeks. BALB-HER2/neu transgenic mice were kindly provided by Dr. Shari Pilon-Thomas (H. Lee Moffitt Cancer Center & Research Institute). All mice were housed at the Animal Research Facility of the H. Lee Moffitt Cancer Center and Research Institute. All mouse studies were reviewed and approved by the Institutional Animal Care and Use Committee at the University of South Florida (#A4100-01). All experiments were performed in strict accordance with the recommendations in the Guide for the Care and Use of Laboratory Animals of the National Institute of Health.

HER2-DC1 s.c. combined with anti-HER2 antibodies treatment in TUBO tumor model

The TUBO cell line expressing rat HER2 was used to induce mouse mammary carcinoma model for HER2^{pos} BC. TUBO cells ($3x10^4$ cells/mouse) were orthotopically injected into the mammary fat pads (MFP) of female BALB/c mice. On day 12 after establishment of tumors, mice were treated with HER2-DC1 s.c. ($1x10^6$ cells/dose, twice weekly for three weeks) and/or anti-HER2 antibodies (clone 7.16.4: 50μg, 7.9.5: 50μg, intraperitoneal (i.p.) injection, once weekly). Control mice were injected with PBS (s.c.,

twice weekly). The multi-epitope MHC class II HER2 peptides pulsed DC1 vaccine (HER2-DC1) was prepared as previously described [29]. For combination therapy, tumor bearing mice received one injection of anti-HER2/neu antibodies on day 12. One week after, mice received HER2-DC1 s.c., concurrently with anti-HER2/neu antibodies. Mice were monitored for tumor growth and tumors were measured twice weekly using digital calipers. Tumor volume was calculated using the formula: volume (mm^3) = length x $width^2 \div 2$.

HER2-DC1 i.t. in combination with anti-HER2 antibodies treatment in TUBO tumor model

After TUBO tumor establishment as described above, mice were randomized into the following groups: (1) untreated, (2) HER2-DC1 i.t., (3) anti-HER2 antibodies (7.16.4+7.9.5), (4) 7.16.4 alone (5) 7.9.5 alone, (6) HER2-DC1 i.t. combined with anti-HER2 antibodies (both clones 7.16.4+7.9.5), (7) HER2-DC1 i.t. combined with 7.16.4 alone. For monotherapy, mice received HER2-DC1 i.t. ($1x10^6$ cells/dose, once weekly for six weeks), anti-HER2 antibodies (7.16.4: 50μg, 7.9.5: 50μg, i.p., once weekly), 7.16.4 alone (100μg, i.p., once weekly) and 7.9.5 alone (100μg, i.p., once weekly). Treatment started on day 12 following tumor injection. For intratumoral (i.t.) delivery, HER2-DC1 ($1X10^6$ cells/50ul of PBS) were drawn into a 1ml syringe using 18G needle and gradually injected into the measurable (4x4mm) palpable tumor directly using a 27G needle. Untreated mice received i.t. injection of sterile PBS. Combination treatment groups 6 and 7 received anti-HER2 antibodies, either both clones 7.16.4+7.9.5 or clone 7.16.4 alone, respectively, on day 12. One week after, mice received HER2-DC1 i.t.

concurrently with anti-HER2 antibodies, either both clones 7.16.4+7.9.5 or clone 7.16.4 alone. Mice were monitored and tumor volumes were measured as described above.

Allogenic HER2-DC1 i.t. or autologous unpulsed DC1 i.t. in combination with anti-HER2 antibodies treatment in TUBO tumor model

After TUBO tumor establishment in BALB/c mice as described above, mice were randomized into the following groups: (1) untreated, (2) autologous unpulsed DC1 i.t., (3) allogenic HER2-DC1 i.t., (4) autologous unpulsed DC1 i.t. combined with anti-HER2 antibodies (both clones 7.16.4+7.9.5) and (5) allogenic HER2-DC1 i.t combined with anti-HER2 antibodies (both clones 7.16.4+7.9.5). Group 2 received autologous unpulsed DC1 i.t. (generated from BALB/c mice and were not pulsed with multi-epitope MHC class II HER2 peptides) once weekly for six weeks. Group 3 received allogenic HER2-DC1 i.t. (generated from C57BL/6 mice and were pulsed with multi-epitope MHC class II HER2 peptides) once weekly for six weeks. Groups 4 and 5 were treated with autologous unpulsed DC1 i.t. or allogenic HER2-DC1 i.t., respectively in combination with anti-HER2 antibodies. Combination treatments were followed as described above. Mice were monitored and tumor volumes were measured.

BALB-HER2/neu transgenic mice treatments and MRI imaging

We utilized BALB-HER2 transgenic mouse model, a classic model of human HER2^{pos} BC, which express rat HER2. The spontaneous focal mammary carcinoma develops in the mammary glands of these mice that slowly progresses from microscopic lesions to invasive tumors. BALB-HER2/neuT mice at 8-9 weeks of age were given HER2-DC1 i.t. (once weekly for

six weeks), anti-HER2/neu antibodies (both clones 7.16.4+7.9.5, i.p., once weekly) or combination of both. HER2-DC1 i.t. delivery was assisted by ultrasound guidance. Mice were examined for spontaneous tumor growth in mammary glands at different timepoints by magnetic resonance imaging (MRI). Mice were anesthetized with 2% isoflurane delivered in 1.5-liter/min oxygen in an induction chamber. Next, mice were transferred and imaged on a 7-Tesla horizontal MRI scanner (Bruker Biospin, Inc. BioSpec AV3HD) using a 35mm Litzcage coil (Doty Scientific). While imaging, ventilation was provided through a nose cone and respiration range was maintained at a range of 40-60 breaths per minute. The core body temperature was monitored and maintained at 37°C by MRI-compatible Small Rodent Heater System (SAII®, SA Instruments, Stony Brook, NY). Anatomical T2-weighted coronal images were acquired using a TurboRARE sequence with field of view (FOV) = 75x35mm^2, echo time/repetition time (TR/TE) = 4513/38ms and slice thickness of 1.2mm and 19 slices.

CT26 hHER2 tumor model and treatments

The CT26 cell line expressing human HER2 was used to induce clinically relevant HER2^{pos} BC and to test the efficacy of HER2-DC1 i.t. combined with anti-HER2 antibodies trastuzumab and pertuzumab treatment or T-DM1. CT26 hHER2 cells (3x10^5 cells/mouse) were injected subcutaneously (s.c.) into female BALB/c mice. On day 7 after establishment of palpable tumors, mice were treated with HER2-DC1 i.t. (every five days), anti-HER2 antibodies (trastuzumab: 90µg, pertuzumab: 90µg, i.p., every five days) and T-DM1 (180µg, i.p., every five days). For combination treatment, mice received single injection of anti-HER2 antibodies (trastuzumab and pertuzumab) or T-

DM1 on day 7. Five days later, mice received HER2-DC1 i.t. concurrently with anti-HER2 antibodies (trastuzumab and pertuzumab) or T-DM1. Tumor growth was monitored and measured twice weekly.

HER2-DC1 combined with anti-HER2 antibodies versus chemotherapy combined with anti-HER2 antibodies in CT26 hHER2 tumor model

After CT26 hHER2 tumor was established as described above, mice were treated with HER2-DC1 i.t. (every five days), anti-HER2 antibodies (trastuzumab: 90µg, pertuzumab: 90µg, i.p., every five days) and paclitaxel (7.5mg/kg, i.p., every three days). For paclitaxel combined with anti-HER2 antibodies treatment, mice received two injections of paclitaxel followed by concurrent treatment with anti-HER2 antibodies (trastuzumab and pertuzumab). HER2-DC1 i.t. and anti-HER2 antibodies combination treatment was carried out as described above.

TUBO bilateral tumor model and treatments

BALB/c mice were injected s.c. with $2.5x10^5$ TUBO cells into both the left and right flank to inoculate primary and distant tumors, respectively. After tumors were palpable, mice were randomized into four groups: (1) untreated, (2) HER2-DC1 i.t., (3) anti-HER2 antibodies (7.16.4+7.9.5) and (4) HER2-DC1 i.t. combined with anti-HER2 antibodies (7.16.4+7.9.5). Treatments were carried out as mentioned above. In groups 2 and 4, HER2-DC1 i.t. was injected only in the primary tumors on the left flank of the mice while the distant tumors on the right flank were left untreated. Tumor size was measured twice a week.

Flow cytometry analysis for host immune cells

After completion of treatments, experimental mice were euthanized, and tumors and spleens were collected under sterile conditions and processed into single-cell suspensions as described previously [29]. Single cell suspensions of tumors were obtained by an enzymatic dissociation method in 10ml of HBSS (Cat. No. MT-21-022-CM, Fisher Scientific), 1mg/ml collagenase (Cat. No. C9891 and C-5138, Millipore Sigma), 0.1mg/ml DNase I (Cat. No. DN25, Millipore Sigma), and 2.5 U/ml of hyaluronidase (Cat. No. H-6254-1G, Millipore Sigma) for 1 hour at 37°C with constant stirring to promote dissociation. Red blood cells were then lysed using ACK lysis buffer for 5 minutes at room temperature. 1x10^6 cells were incubated with Live/Dead Zombie near IR (Cat. No. 423106, BioLegend, San Diego, CA) for 30 minutes at room temperature in the dark. Cells were then washed with PBS, resuspended in FACS buffer, and stained with lymphoid immune cell phenotyping antibodies anti-CD45 BUV 395 (Cat. No. 564279, Clone 30-F11, BD Biosciences, San Jose, CA), anti-CD3 APC (Cat. No. 553066, Clone 145-2C11, BD Biosciences), anti-CD4 PerCP-Cy5.5 (Cat. No. 550954, Clone RM4-5, BD Biosciences), anti-CD8 Pac Blue (Cat. No. 558106, Clone 53–6.7, BD Biosciences), anti-CD44 FITC (Cat. No. 553133, Clone IM7, BD Biosciences), anti-CD62L BUV737 Cat. No. 612833, Clone MEL-14, BD Biosciences), anti-CD19 PE/Cyanine7 (Cat. No. 115520, Clone 6D5, BioLegend), and anti-CD49b or DX-5 pan NK PE (Cat. No. 108908, Clone DX5, BioLegend) or myeloid cells antibodies anti-CD45 BUV395 (Cat. No. 564279, Clone 30-F11, BD Biosciences), anti-Gr1 or Anti-Ly-6G and Ly-6C APC (Cat. No. 553129, Clone RB6-8C5, BD Biosciences), anti-CD11b PerCP-Cy5.5 (Cat. No. 550993, Clone M1/70, BD Biosciences), anti-CD11c PE (Cat. No. 117308, Clone N418, BioLegend), anti-F4/80 PE/Cyanine7 (Cat. No. 123114, Clone

BM8, BioLegend), anti-CD80 Pac Blue (Cat. No. 104724, Clone 16-10A1, BioLegend), anti-IA^d FITC (Cat. No. 115006, Clone 39-10-8, BioLegend) and anti-CD206 BV650 (Cat. No. 141723, Clone C086C2, BioLegend) for 30 minutes on ice. After washing with PBS, flow cytometry was performed on an LSRII cytometer (BD Biosciences) and FACS data was analyzed using FlowJo software (FlowJo™, RRID:SCR_008520).

In vivo HER2-DC1 trafficking analysis

HER2-DC1 (1x10^6 cells) was labeled with CellTrace Violet (Cat. No. C34557, Invitrogen) following manufacturer's instructions and injected into the established TUBO bilateral tumor model. All treatment conditions were followed as described above. Tumors from treated and untreated flanks and tumor-draining lymph nodes (TDLNs) were harvested after 24 and 48 hours of treatment. Following incubation with Live/Dead Zombie near IR, single cell suspensions were stained with a HER2-DC1 trafficking panel CellTrace Violet, anti-CD45 FITC (Cat. No. 11-0451-82, Clone 30-F11, Fisher Scientific), anti-CD11c BV785 (Cat. No. 117336, Clone N418, BioLegend), anti-MHCII PE (Cat. No. 50-5321-U100, Clone M5/114.15.2, Tonbo Biosciences, San Diego, CA) and anti-CD103 APC (Cat. No. 121414, Clone 2E7, BioLegend) or intratumoral DC subsets antibodies anti-CD45 FITC (Cat. No. 11-0451-82, Fisher Scientific), anti-MHCII PE (Cat. No. 50-5321-U100, Tonbo Biosciences), anti-Ly-6G/Ly-6C (Gr-1) PE/Dazzle (Cat. No. 108452, Clone RB6-8C5, BioLegend), anti-CD103 APC (Cat. No. 121414, BioLegend), anti-F4/80 Alexa Fluor 700 (Cat. No. 123130, Clone BM8, BioLegend), anti-CD11b PerCP-Cy5.5 (Cat. No. 550993, Clone M1/70, BD Biosciences), and anti-CD11c BV785 (Cat. No. 117336, Clone N418, BioLegend) and analyzed by flow cytometry as described

above. Cells were also stained to identify apoptotic cells using APC Annexin V Apoptosis Detection Kit with 7-AAD (Cat. No. 640930, BioLegend) and analyzed using a LSRII or Canto cytometer (BD Biosciences) with FlowJo software (FlowJo™, RRID:SCR_008520).

IFN-γ quantification by ELISA

Single cell suspensions from spleens and inguinal lymph nodes were prepared as described above. To examine the anti-HER2 Th1 immune response generated following treatments, splenocytes ($2x10^6$ cells) were stimulated with or without multi-epitope MHC class II rat HER2/neu peptides p5 (ELAAWCRWGFLLALLPPGIAG; 2μg/ml), p435 (IRGRILHDGAYSLTLQGLGIH; 2μg/ml) and p1209 (SPPHPSPAFSPAFDNLYYWDQ; 2μg/ml) individually for 72 hours [29]. In addition, TDLNs and non-draining lymph nodes (NDLNs) ($1x10^5$ cells) were co-cultured for 72 hours with or without HER2-DC1 ($1x10^4$ cells) individually pulsed with p5, p435 and p1209. Following incubation, culture supernatants were collected, and floating cells were removed by centrifugation at 1000rpm for 5 minutes. IFN-γ secretion was measured using a commercially available mouse IFN-γ Quantikine ELISA kit (Cat. No. SMIF00, R&D Systems, Minneapolis, MN) following manufacturer's instructions.

Cytokine and chemokine arrays

Various Th cytokines (IFN-γ, TNF-α, IL-2, IL-4, IL-6, IL-10, IL-17A and IL-21) and proinflammatory chemokines (RANTES/CCL5, TACR/CCL17, KC/CXCL1, MIG/CXCL9, IP-10/CXCL10, MIP-1α/CCL3 and MDC/CCL22) in serum samples of experimental mice

were measured using LEGENDplex mouse Th cytokine panel detection (Cat. No. 740073, BioLegend), and LEGENDplex mouse proinflammatory chemokine panel detection (Cat. No. 740074, BioLegend) according to the manufacturer's recommendations.

Immunohistochemistry

Immunohistochemistry was performed as previous described [25]. Briefly, tumor tissues were collected from the experimental mice, fixed in formalin, and embedded in paraffin. Five micrometer thick paraffin tumor tissue sections were deparaffinized with xylene and rehydrated in graded ethanol. Antigen retrieval was performed using Tris-EDTA buffer and slides were incubated with 3% hydrogen peroxidase for 30 minutes to block endogenous peroxidase activity. Slides were then washed and incubated with 10% normal goat serum in Tris-buffered saline (TBS). Thereafter, slides were incubated with anti-rabbit HER2 antibody (Cat. No. 4290S, Cell Signaling Technologies, Danvers, MA) for overnight. Slides were washed and incubated with secondary antibody labeled with HRP for 1 hour at 37°C. The color development was detected using 3,3'-diaminobenzidine (DAB) and the slides were counter-stained with hematoxylin. Slides were scanned using a Leica Aperio™ AT2 scanner (Vista, CA) at the Microscopy Core Facility at H. Lee Moffitt Cancer Center and Research Institute.

$CD4^+$ and $CD8^+$ T cell depletion experiments

Anti-CD4 and anti-CD8 antibodies were used to deplete host $CD4^+$ and $CD8^+$ T cells. Mice were injected with or without 300 ug of depleting antibody (i.p.) twice a week beginning three days prior to TUBO cells ($3x10^4$ cells/mouse) inoculation in the MFP

and was continued until the endpoint. Non-depleted mice were injected i.p. with rat IgG2b isotype antibody. After establishment of TUBO tumors, mice were treated as described above. Tumor volume was measured twice a week.

FcγR KO mice model experiment

FcγR-deficient C.129P2(B6)-*Fcer1g*^{tm1Rav} N12 female mice were injected with TUBO cells (3x10^{4}) orthotopically into the MFP. TUBO tumor bearing mice were treated with HER2-DC1 i.t., anti-HER2 antibodies, or a combination of both as described above.

Western blot analysis

Whole protein extracts from tumor samples were prepared by homogenization in RIPA buffer (Cat. No. 20-188, Millipore, Billerica, MA) mixed with protease inhibitor cocktail (Cat. No. P8340-1ML, Sigma-Aldrich, St. Louis, MO) and phosphatase inhibitor (Cat. No. A32957, Pierce) and incubated for 20 minutes at 4°C. After centrifugation at 14,000rpm for 20 minutes at 4°C, supernatants were collected, and protein concentration was measured by Bradford protein assay (Cat. No. 5000006, Bio-Rad, Hercules, CA). 20-40 µg of protein were run on a 4-12% SDS-PAGE gel (GenScript, Piscataway, NJ) and transferred onto PVDF membranes (Cat. No. IPVH00010, Millipore) using eBlot® L1 wet transfer system (GenScript). The membranes were incubated in 5% (m/v) BSA prepared in Tris-buffered saline buffer-Tween (TBST) and probed with rabbit anti-HER2 (Cat. No. 2165S, Cell Signaling Technologies), rabbit anti-pHER2 Tyr1248 (Cat. No. 2244S, Cell Signaling Technologies), rabbit anti-pHER2 Tyr1221/1222 (Cat. No. 2243S, Cell Signaling Technologies), rabbit anti-pHER2 Tyr877

(Cat. No. 2241S, Cell Signaling Technologies), rabbit anti-pSTAT1 Tyr701(Cat. No. 9167S, Cell Signaling Technologies), rabbit anti-pSTAT1 Ser727 (Cat. No. 9177S, Cell Signaling Technologies), rabbit anti-pSTAT3 Tyr705 (Cat. No. 9145S, Cell Signaling Technologies), rabbit anti-pSTAT5 Tyr694 (Cat. No. 9351S, Cell Signaling Technologies), rabbit anti-p-P38 MAPK (Thr180/Tyr182; Cat. No. 4511S, Cell Signaling Technologies), rabbit anti-pERK1/2 (Thr202/Tyr204; Cat. No. 9101S, Cell Signaling Technologies), rabbit anti-pAkt (Ser473; Cat. No. 9271S, Cell Signaling Technologies), rabbit anti-PI3K (Cat. No. 4257S, Cell Signaling Technologies), rabbit anti-pJAK2 (Tyr1007/1008; Cat. No. 3776S, Cell Signaling Technologies), rabbit anti-cleaved caspase 3 (Asp175; Cat. No. 9661S, Cell Signaling Technologies), rabbit anti-caspase 3 (Cat. No. 9662S, Cell Signaling Technologies), rabbit anti-cyclin D1 (Cat. No. 2978S, Cell Signaling Technologies), mouse anti-p15 (Cat. No. sc-377412, Santa Cruz Biotechnology, Dallas, TX), rabbit anti-p16 (Cat. No. ab211542, Abcam, Boston, MA) and rabbit anti-β actin (Cat. No. 4967S, Cell Signaling Technologies) antibodies. After washing with TBST, membranes were incubated with goat anti-rabbit IgG HRP (Cat. No. 7074S, Cell Signaling Technologies) or goat anti-mouse IgG HRP (Cat. No. 1721011, Bio-Rad, Hercules, CA) secondary antibodies for one hour at room temperature. Bands were visualized with the Pierce ECL Western Blotting Substrate (Cat. No. 32106, ThermoFisher Scientific) and imaged on an Odyssey Imaging System (LI-COR Biosciences, Lincoln, Nebraska, USA).

Statistical analysis

All data were expressed as mean ± standard error of the mean (SEM). The mean differences of several groups were analyzed using one way analysis of variance (ANOVA) with Tukey's multiple comparisons post hoc test. The mean differences between two groups were analyzed using an unpaired or paired two-tailed t test. Survival curves were analyzed using the Log-rank (Mantel-Cox) test or Gehan-Breslow-Wilcoxon test. The statistical analyses were performed using GraphPad Prism 8. Data with p value <0.05 were considered statistically significant.

Supplemental figures

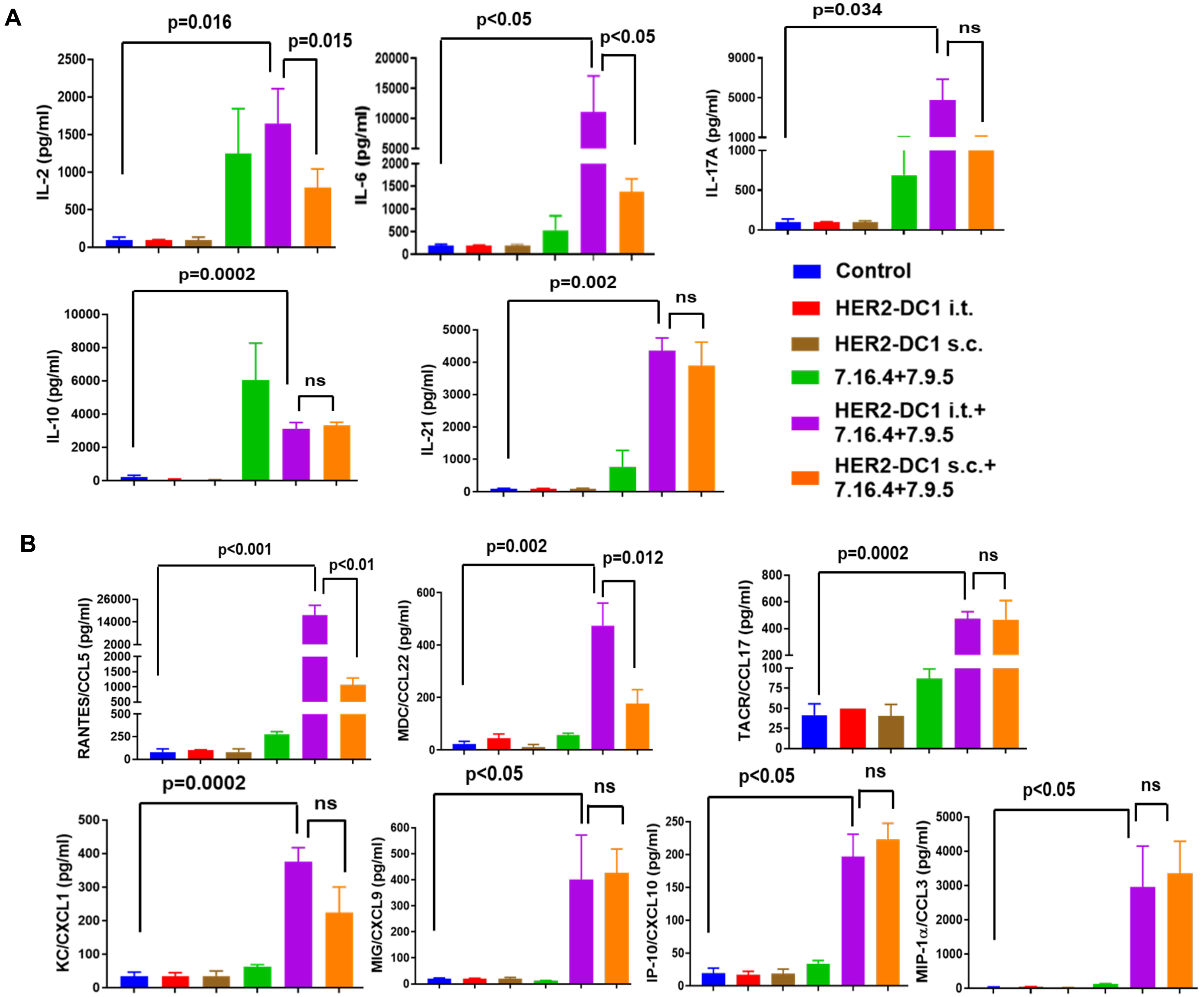

Supplemental Figure 1 Serum level of Th1 cytokines and chemokines.
(A) Th1 cytokines IL-2, IL-6, IL-17A, IL-10 and IL-21 levels in the serum of TUBO tumor bearing mice receiving different treatments as indicated. (B) Proinflammatory chemokines RANTES/CCL5, TACR/CCL17, KC/CXCL1, MIG/CXCL9, IP-10/CXCL10, MIP-1α/CCL3 and MDC/CCL22 levels in the serum of TUBO tumor bearing mice that received different treatments as indicated. Mean ± SEM. i.t., intratumoral; s.c., subcutaneous; ns., not significant.

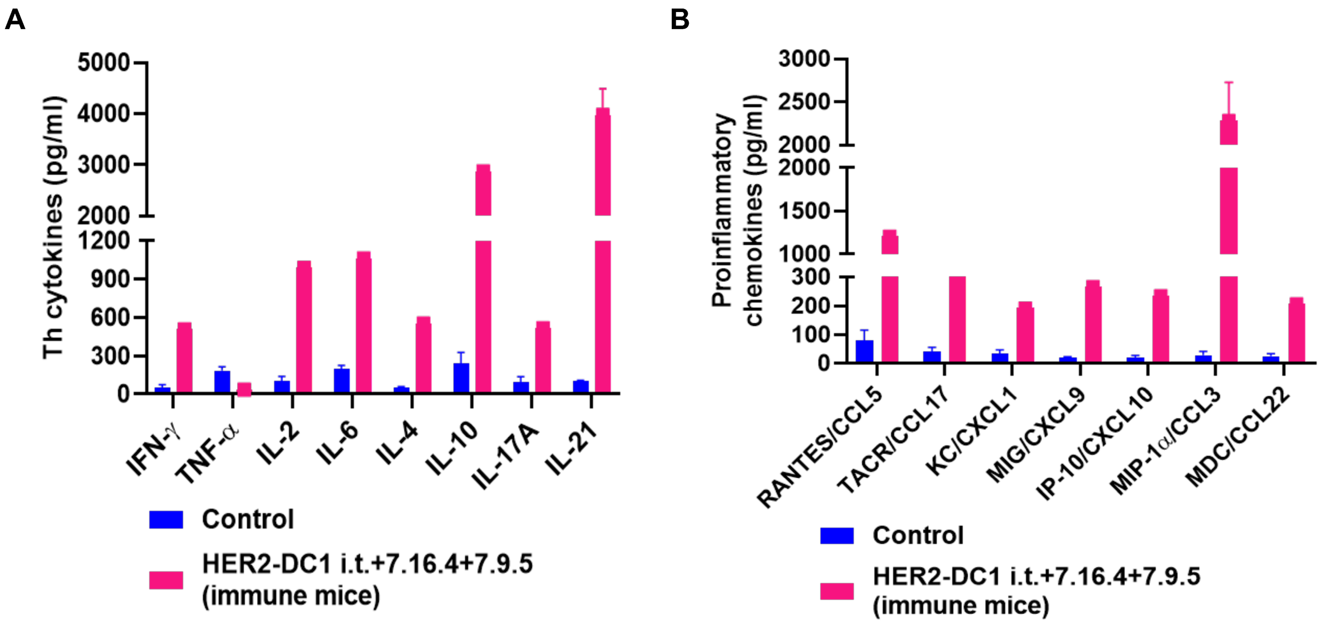

Supplemental Figure 2 Serum level of Th1 cytokines and chemokines in immune mice that are cured by HER2-DC i.t in combination with anti-HER2 antibodies treatment and rejected secondary TUBO tumor challenge.
(A) Th cytokines IFN-γ, TNF-α, IL-2, IL-4, IL-6, IL-10, IL-17A and IL-21 in the serum of immune mice. (B) Proinflammatory chemokines RANTES/CCL5, TACR/CCL17, KC/CXCL1, MIG/CXCL9, IP-10/CXCL10, MIP-1α/CCL3 and MDC/CCL22 levels in the serum of immune mice.

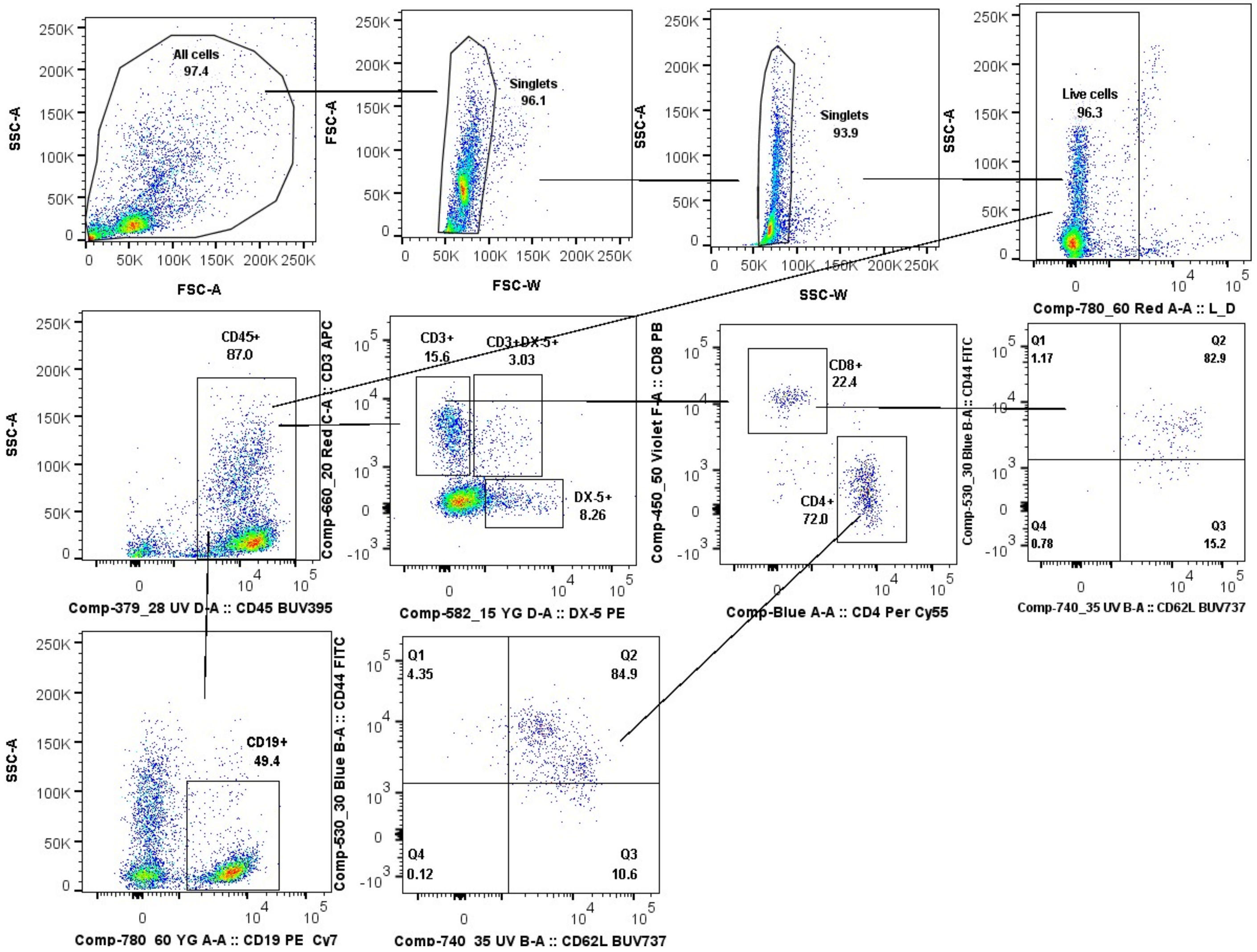

Supplemental Figure 3 Gating strategy for identifying CD3+CD4+ T cells, CD4$^+$CD44$^+$CD62L$^-$ effector memory cells, CD4$^+$CD44$^+$CD62L$^+$ central memory cells, CD4$^+$CD44$^-$CD62L$^-$ effector cells, CD3+CD8+ T cells, CD8$^+$CD44$^+$CD62L$^-$ effector memory cells, CD8$^+$CD44$^+$CD62L+ central memory cells, CD8$^+$CD44$^-$ CD62L$^-$ effector cells, CD3+DX5+(CD49b+) NKT cells, DX5+(CD49b+) CD3- NK cells and CD19+ B cells among CD45+ cells in the tumors of experimental groups.

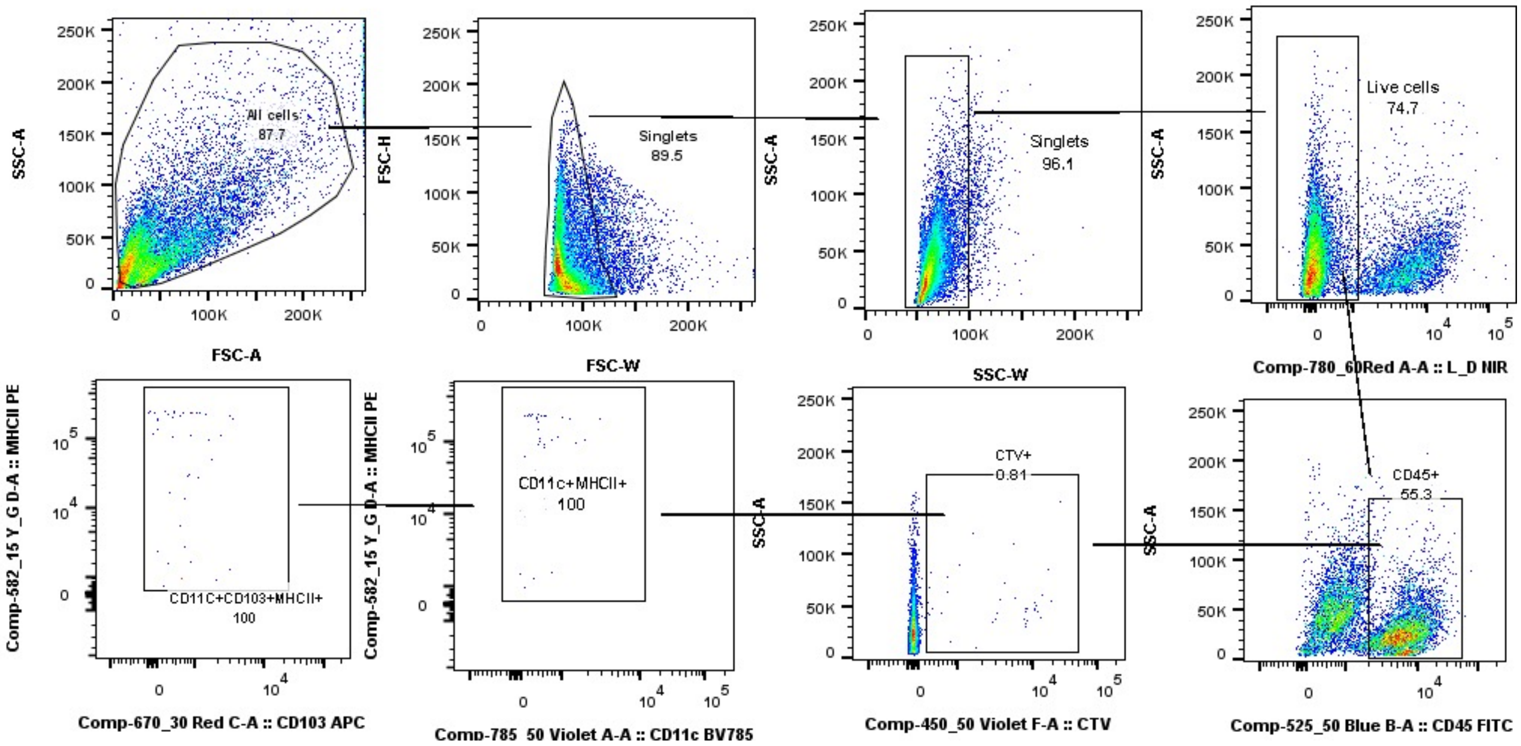

Supplemental Figure 4 Gating strategy for identifying CellTrace Violet labeled HER2-DC1 (CD45⁺CellTrace Violet⁺CDIIc⁺MHCII⁺CD103⁺) in the treated primary tumors and untreated distant tumors and TDLNs of experimental groups. CTV., CellTrace Violet.

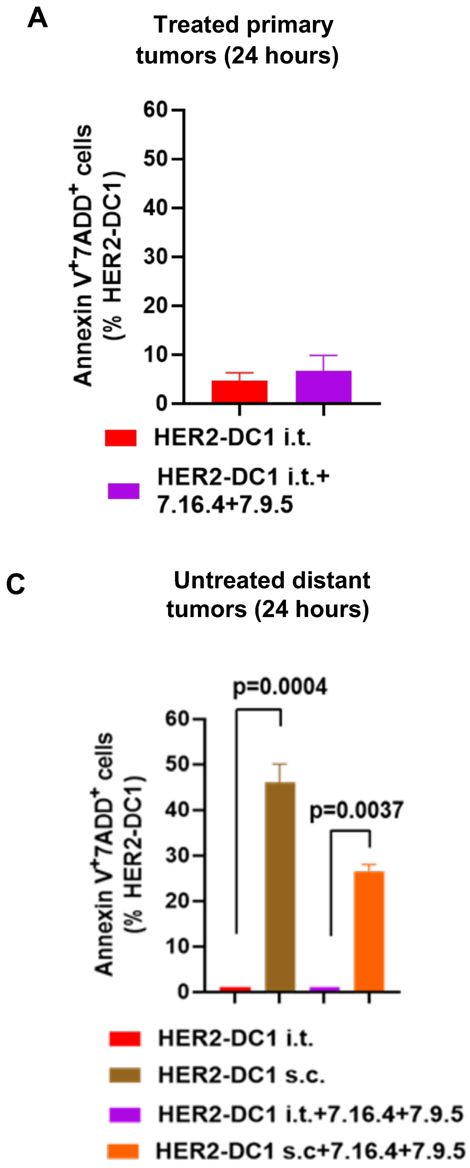

Supplemental Figure 5 Detection of apoptotic cells among the migrated CellTrace Violet labeled HER2-DC1. (A, B) The percent apoptotic cells among the detected CellTrace Violet labeled HER2-DC1 in treated primary tumors at 24 and 48 hours was analyzed by flow cytometry. (C, D) The percent apoptotic cells among the migrated CellTrace Violet labeled HER2-DC1 in untreated distant tumors at 24 and 48 hours was analyzed by flow cytometry.

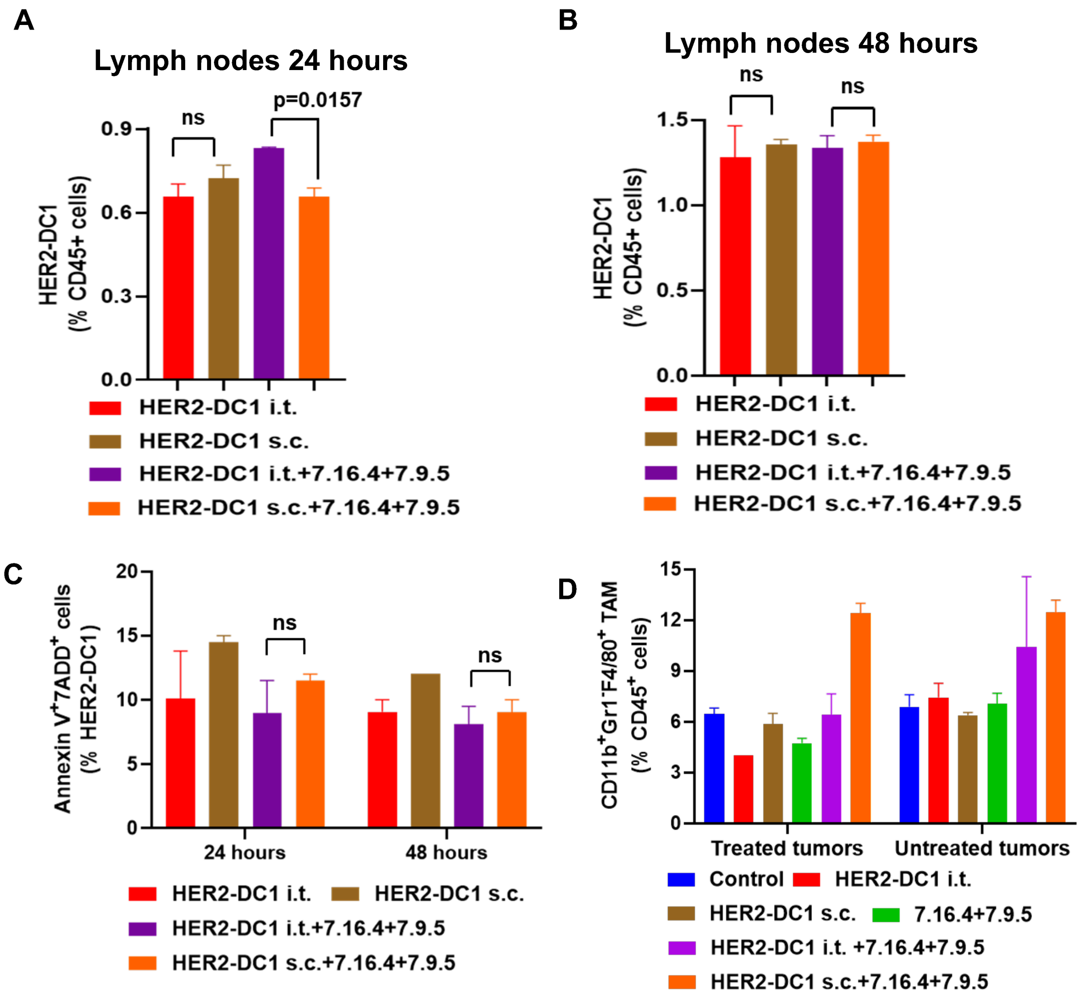

Supplemental Figure 6 (A, B) level of migrated CellTrace Violet labeled HER2-DC1 in TDLNs of experimental groups at 24 and 48 hours was analyzed by flow cytometry. (C) The percent apoptotic cells among the migrated CellTrace Violet labeled HER2-DC1 in TDLNs at 24 and 48 hours. (D) TAM level in the TME of treated primary tumors and untreated distant tumors was analyzed by flow cytometry. TDLNs., tumor draining lymph nodes; TAM., tumor associated macrophages; TME., tumor microenvironment; ns., not significant.

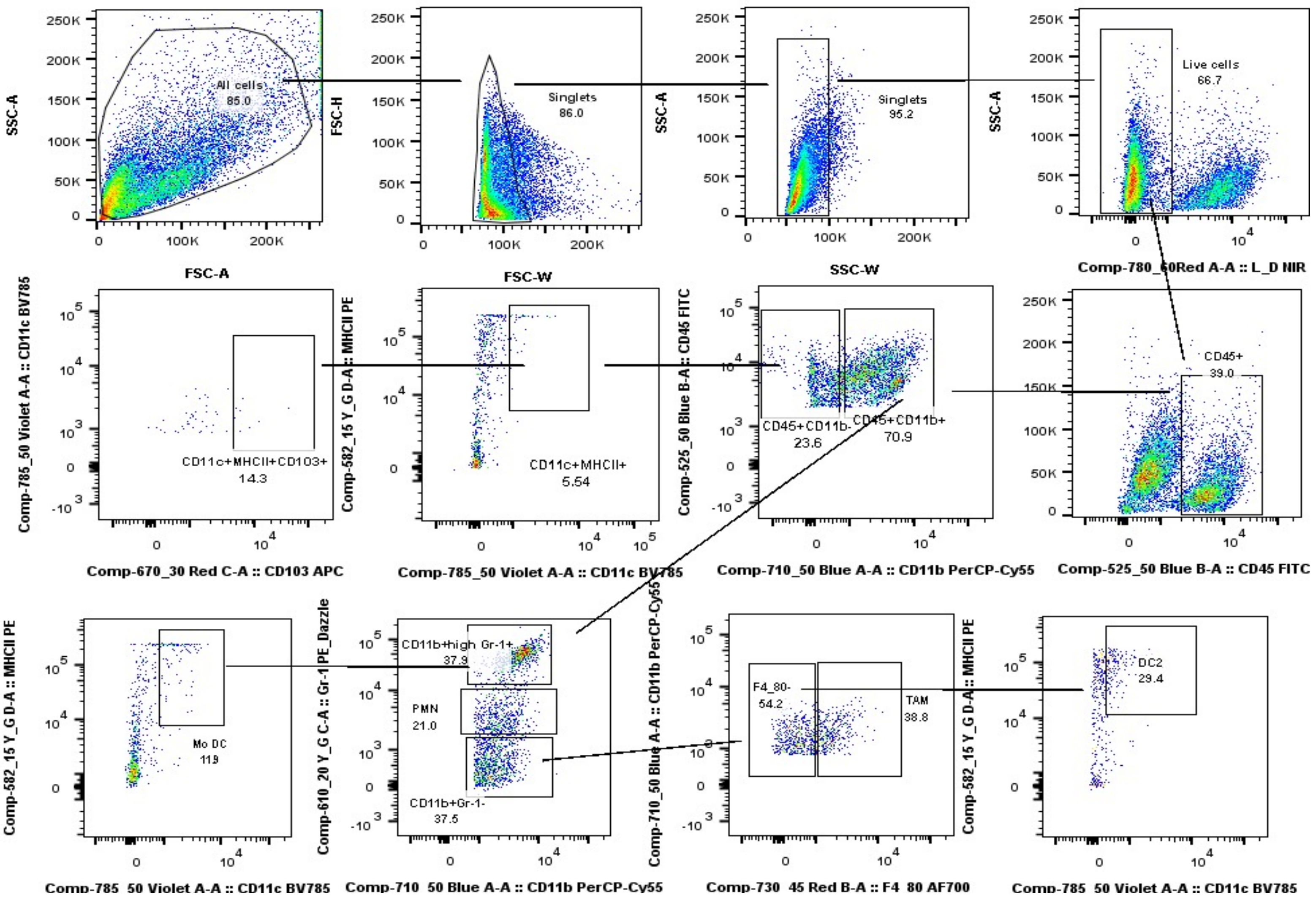

Supplemental Figure 7 Gating strategy for identifying cDC1 ($CD45^{+}CD11c^{+}CD11b^{-}MHCII^{+}CD103^{+}$), cDC2 ($CD45^{+}CD11b^{+}Gr1^{-}F4/80^{-}CD11c^{+}MHCII^{+}$), MoDCs ($CD45^{+}CD11b^{+}Gr1^{+high}CD11c^{+}MHCII^{+}$) and TAM ($CD45^{+}CD11b^{+}Gr1^{-}F4/80^{+}$) in the treated primary tumors and untreated distant tumors of experimental groups. cDC1., conventional DC1; cDC2., conventional DC2; MoDCs., monocytic DCs; TAM., tumor associated macrophages.

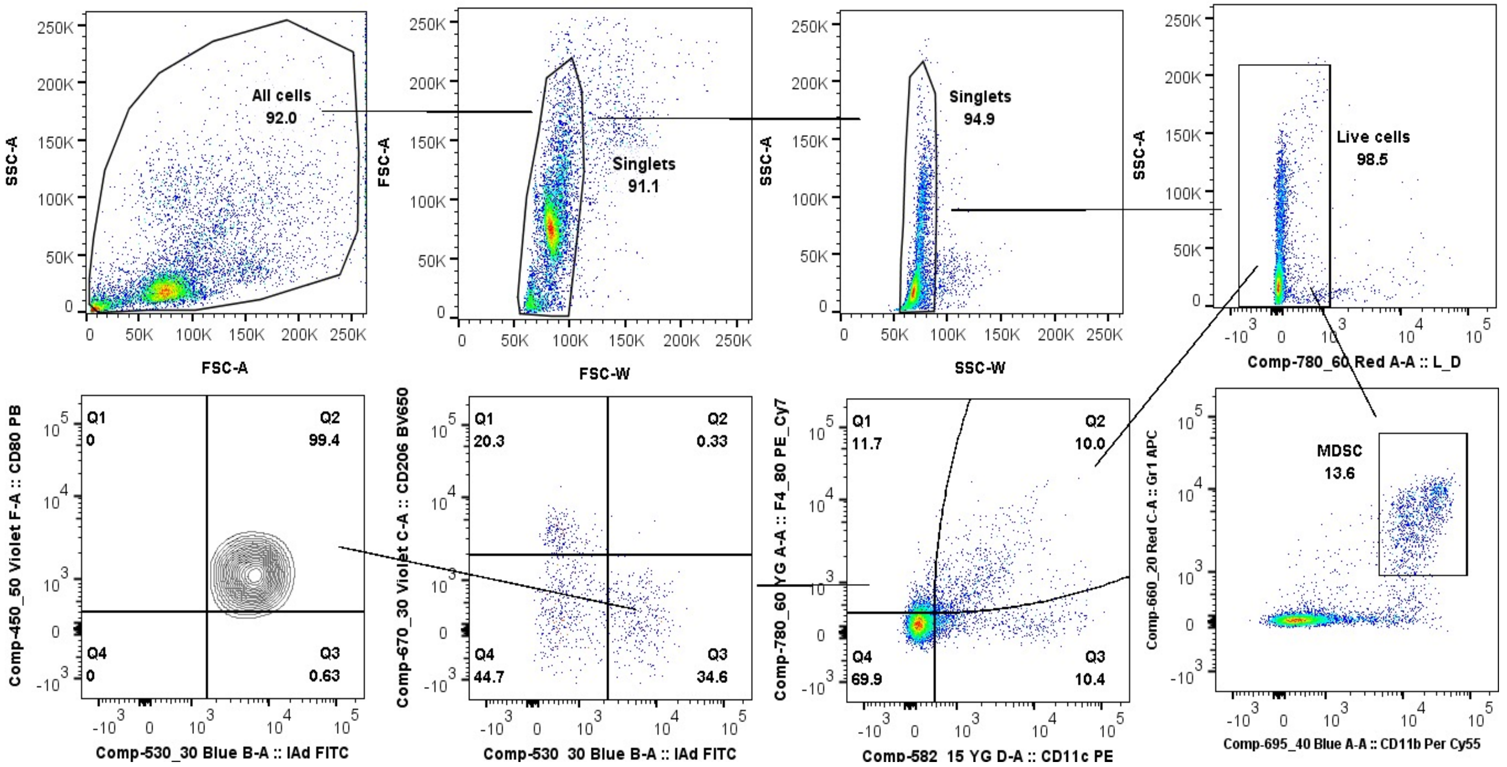

Supplemental Figure 8 Gating strategy for identifying MDSCs (CD11b+Gr1+), M1 (F4/80+IAd+CD80+CD206-) macrophages and M2 (F4/80+CD206+) macrophages among CD45+ cells in the tumors of experimental groups.

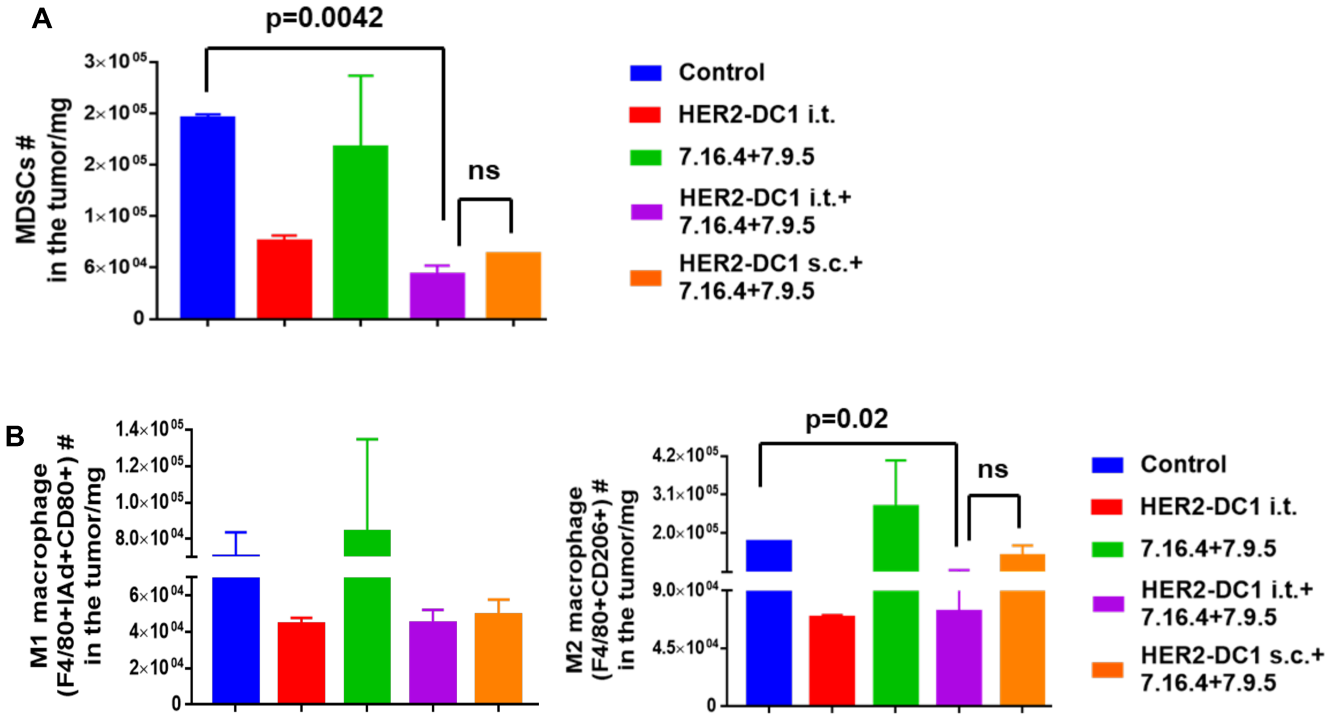

Supplemental Figure 9 Effect of HER2/neu-DC1 in combination with anti-HER2/neu antibodies treatment on MDSCs and M1/M2 macrophages. (A) MDSCs level in the tumors of experimental groups was analyzed by flow cytometry. Mean ± SEM. Control versus HER2/neu-DC1 i.t.+7.16.4+7.9.5 (p=0.0042). HER2/neu-DC1 s.c.+7.16.4+7.9.5 versus HER2/neu-DC1 i.t.+7.16.4+7.9.5 (ns). (B) Level of M1 and M2 macrophages in the tumors of experimental groups was analyzed by flow cytometry. Mean ± SEM. Control versus HER2/neu-DC1 i.t.+7.16.4+7.9.5 (p=0.02). HER2/neu-DC1 s.c.+7.16.4+7.9.5 versus HER2/neu-DC1 i.t.+7.16.4+7.9.5 (ns). i.t., intratumoral; s.c., subcutaneous; ns., not significant.

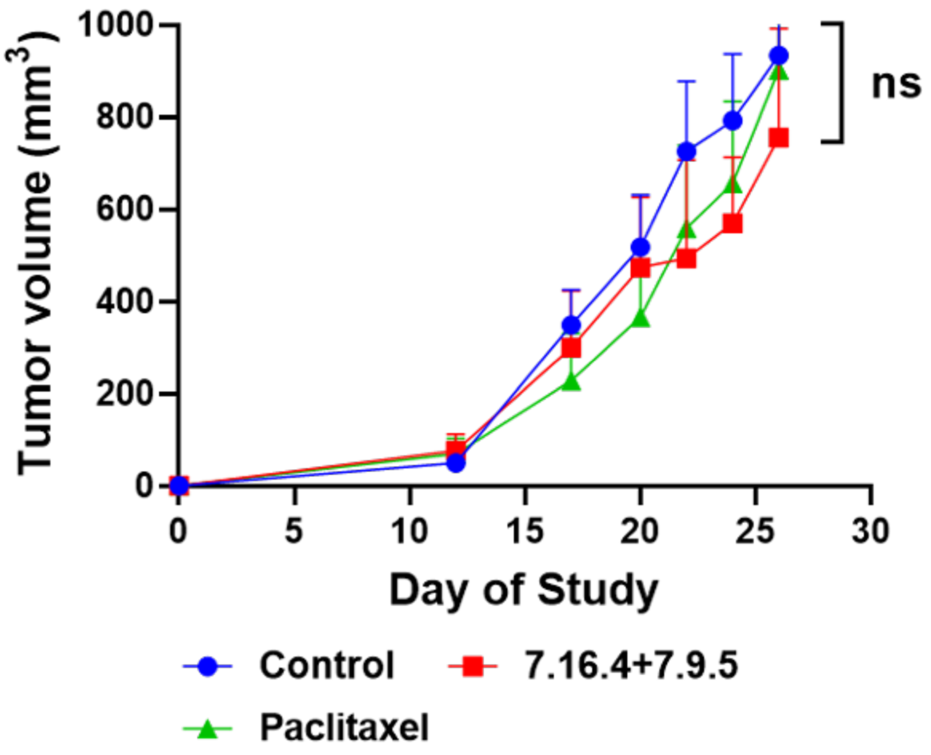

Supplemental Figure 10 Effect of chemotherapy paclitaxel in HER2^{pos} TUBO tumor model. BALB/c mice bearing TUBO tumors were treated with paclitaxel or anti-HER2 antibodies ((both clones 7.16.4+7.9.5) or left untreated as described in methods. Tumor growth was monitored two time a week (n=6). ns., not significant.

Graphical abstract

Intratumoral delivery of dendritic cells plus anti-HER2 therapy triggers both robust systemic antitumor immunity and complete regression in HER2 mammary carcinoma

Authors
Ganesan Ramamoorthi, Krithika N. Kodumudi, Colin Snyder, Payal Grover, Hongtao Zhang, Mark I. Greene, Amrita Basu, Corey Gallen, Doris Wiener, Ricardo L.B. Costa, Hyo S. Han, Gary Koski, Brian J. Czerniecki

Correspondence
brian.czerniecki@moffitt.org

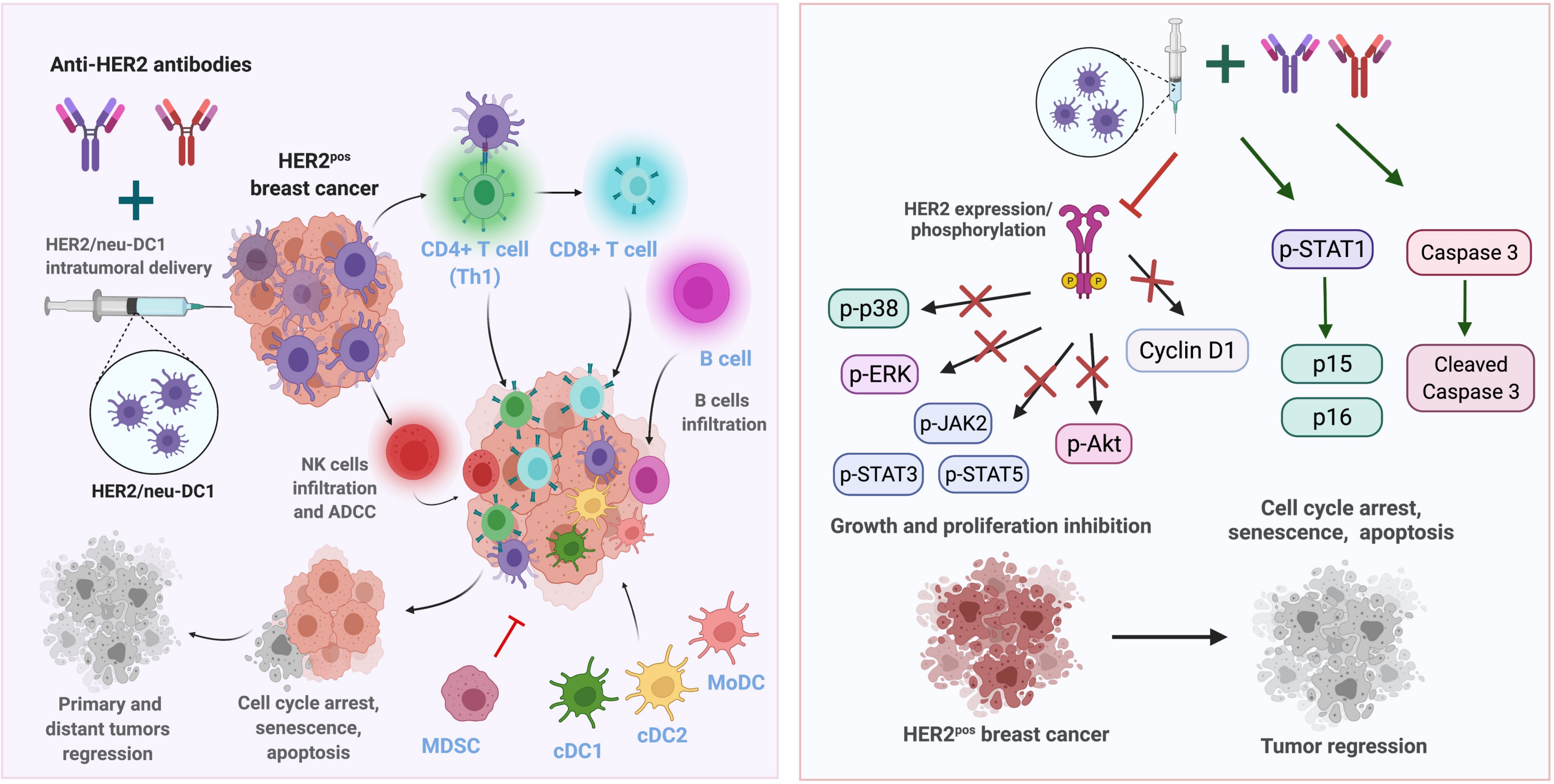

In Brief

Intratumoral delivery of HER2-DC1 plus anti-HER2 antibodies generated robust systemic anti-tumor immunity which rendered tumor regression of treated tumors as well as untreated distant tumors and effectively modulated HER2 oncogenic signaling pathways in HER2 breast cancer. This combination treatment was more effective than standard chemotherapy with anti-HER2 antibodies.
